# Qualitative exploration of gambling harm among UK veterans: normalisation, stigma and postservice escalation

**DOI:** 10.1136/bmjopen-2025-109458

**Published:** 2026-03-25

**Authors:** Dana Dekel, Adanma Ekenna, Hillary Engward, Lauren R Godier-McBard, Chris Kay, Thomas Kersey, Matt Fossey, Simon Dymond

**Affiliations:** 1 Centre for Military Gambling Research, Swansea University, Swansea, UK; 2 Anglia Ruskin University Veterans & Families Institute for Military Social Research, Chelmsford, UK; 3 Department of Psychology, Reykjavík University, Reykjavík, Iceland

**Keywords:** MENTAL HEALTH, QUALITATIVE RESEARCH, Behavior

## Abstract

**Abstract:**

**Objective:**

This study explored the lived experiences of UK Armed Forces veterans affected by gambling-related harm. It examined how military culture, institutional practices and life transitions shaped gambling behaviours, barriers to help-seeking and the long-term impact on well-being. While quantitative research has documented elevated gambling-related harm among veterans, little qualitative work has examined how veterans themselves understand, experience and navigate gambling harm across military and post-military contexts.

**Design:**

Reflexive thematic analysis of one-to-one semistructured interviews, covering topics such as the nature of participants’ gambling activities, the impact on their lives, pathways to gambling behaviours and help-seeking.

**Participants:**

Participants were UK veterans (n=14), aged 31–60, including one female, from three service branches, all of whom self-identified as having experienced gambling-related harm.

**Results:**

Four interrelated themes were generated: (1) Gambling as normalised in both civilian and military contexts, reinforced by institutional routines and downtime activities; (2) Gambling as an emotional coping mechanism, shaped by institutional norms of stoicism and emotional control; (3) Stigma, silence and structural barriers to help-seeking, including fears of professional repercussions and (4) Escalation postdischarge, driven by isolation, unstructured time, digital gambling access and difficulties adjusting to civilian life. Participants reported concealment, debt and relational breakdowns, with some disclosing suicidal ideation linked to gambling harm.

**Conclusions:**

Gambling harm among UK veterans is shaped by a complex interplay of cultural, emotional and institutional factors. While gambling opportunities are embedded in military life, systems of support remain inconsistent and often punitive. Gambling remains under-recognised as a serious mental health issue within military and veteran care pathways. Findings highlight the need for stigma-free, culturally informed interventions across the military life cycle, including routine screening, gambling harm education and trauma-informed care.

**STRENGTHS AND LIMITATIONS OF THIS STUDY**

- This study used a descriptive qualitative approach enabled rich, contextualised insights into a complex and sensitive topic, while remaining grounded in participants’ own language and meanings.
- Involvement of a lived experience panel in piloting the interview guide enhanced the cultural sensitivity and clarity of the questions, improving the overall relevance and accessibility of the data collection process.
- Purposive sampling ensured the inclusion of participants from different service branches, ranks and lengths of service, allowing for a diverse range of perspectives.
- The sample consisted only of veterans who self-identified as having experienced gambling harm, which may exclude those with less insight or differing experiences, potentially limiting the breadth of perspectives captured.
- While the sample was diverse in terms of military background, most participants were male and white British, which may limit the transferability of findings to more demographically varied veteran populations.

## Introduction

 Gambling-related harm is a significant public health issue globally, with far-reaching consequences for individuals, families and communities.^1^ Gambling-related harm is understood as a multidimensional concept that extends beyond gambling behaviour itself to encompass harms to financial resources, physical and mental health, social and family relationships, and occupational functioning.^1 2^ These frameworks emphasise that harm may occur across a continuum of gambling involvement and can affect both individuals who gamble and those around them. In the UK, Armed Forces veterans are disproportionately affected, exhibiting substantially higher rates of gambling-related harm compared with the general population.^3^ Research indicates that veterans are approximately seven times more likely to experience problem gambling (PG), defined as a pattern of gambling behaviour that disrupts personal, familial or occupational functioning, with 43% of veterans reporting gambling issues compared with just 6.5% of non-veterans.^4^

Several factors contribute to this elevated risk. Veterans are uniquely vulnerable to risky behaviours, including PG, due to their exposure to trauma and the profound cultural shifts they undergo following military service.^5^ Military life is governed by strict cultural norms,^6^ characterised by highly specialised roles with limited flexibility,^7^ frequent exposure to challenging and potentially dangerous environments, and for some personnel, direct combat. Sustained periods in high-stress operational contexts further differentiate military roles from civilian employment.^8^ On leaving military service, veterans face significant adjustment challenges, such as loss of structure and camaraderie, difficulty finding meaningful civilian employment and managing physical or psychological health problems, which may contribute to heightened engagement in high-risk behaviours, including gambling.^9^,^12^

Military life fosters a distinctive behavioural culture shaped by hierarchy, routine and risk exposure. Social norms within this environment often promote emotional suppression and competitiveness, reinforcing a masculine ideal that can normalise high-risk behaviours and deprioritise emotional well-being.^13^ Within military institutions, dominant constructions of masculinity emphasise stoicism, endurance and self-reliance, while expressions of vulnerability and emotional distress are frequently stigmatised as incompatible with the ideal soldier identity.^14^ This cultural framework may inadvertently desensitise personnel to signs of emerging harm and discourage early recognition of problematic gambling. Fox and Pease^14^ further argue that these norms shape how distress is interpreted and managed, often privileging avoidance, emotional containment and risk-taking over help-seeking or early intervention. Additionally, many veterans face identity confusion, social isolation and economic instability during reintegration, all of which may exacerbate susceptibility to gambling problems.^15^

This heightened vulnerability is further compounded by the prevalence of co-occurring mental health conditions such as post-traumatic stress disorder, depression and substance use disorders, which are significantly more common among veterans than in the general population.^16^,^19^ These comorbidities not only increase the risk of developing PG but may also complicate recognition, diagnosis and treatment.^20^ Despite this elevated risk, gambling-related harm is not systematically assessed within UK military or veteran healthcare systems. Serving personnel are not routinely screened for gambling during pre-enlistment, active duty or at discharge, revealing a critical oversight in prevention and care pathways.^21^ Recent analysis confirms the absence of validated, military-specific screening tools for gambling, and no consensus exists on appropriate measures tailored for service populations.^22^ These gaps are compounded by recommendations from Dighton *et al*,^23^ urging the integration of routine gambling assessment for current and former military personnel to enable early detection and intervention.

Champion *et al*^24^ found that serving UK Armed Forces personnel encounter multiple structural and psychosocial obstacles when attempting to access support for gambling problems. These include a pervasive stigma surrounding mental health and addiction, limited awareness of support services and insufficient integration of gambling screening within routine military healthcare pathways. Further compounding this is a culture that valorises stoicism, self-discipline and endurance, which may lead to the minimisation or concealment of distress and delay help-seeking until crises emerge.^13 19^ These findings align with international literature, which highlights similar patterns among veterans in other jurisdictions,^17 18^ pointing to systemic challenges in identifying and addressing gambling harm early.

Concurrently, the normalisation of gambling within both civilian and military contexts plays a powerful role in shaping behaviour and risk. In the UK, gambling is a culturally sanctioned and heavily marketed activity, with high levels of public exposure across sports, online platforms and advertising.^1^ Military environments may further entrench permissive attitudes toward gambling, with activities often embedded in recreation and social rituals during deployment and downtime.^3 11^ These practices may be interpreted by personnel as benign or even endorsed, which can mask escalating harm and delay recognition of problematic behaviour.^24^ As such, the line between recreational and harmful gambling may become increasingly blurred, particularly when gambling is viewed as a routine or stress-relieving activity in high-intensity operational environments.^6^

Veterans’ gambling motivations are also shaped by a complex interplay of personal, psychological and situational factors. Studies have shown that gambling is often used as a coping strategy to manage boredom, emotional distress or trauma-related symptoms, especially among those exposed to combat or operational stress.^10 16^ Preferences may shift over time, with veterans gravitating toward fast-paced, high-reward forms of gambling, such as online betting or slot machines, which offer temporary distraction or emotional regulation but are associated with a higher risk of addiction.^11^ These patterns may intensify over the course of a military career or following exposure to trauma, suggesting a progression from socially motivated to more compulsive gambling behaviour.

The post-military transition represents a particularly high-risk period for the development or escalation of gambling harm. Loss of routine, camaraderie and identity, combined with civilian stressors such as unemployment, housing insecurity or relationship breakdowns, may leave veterans vulnerable to maladaptive coping strategies.^5 15^ The disconnection from military support networks and the challenges of reintegration can amplify feelings of isolation and purposelessness, further entrenching gambling behaviours.^7 10^ Veterans often report that gambling provides a means to fill time or regain a sense of control, yet the consequences, including debt, shame and family strain, can be profound and enduring.^4^

While quantitative studies have mapped the prevalence and correlates of gambling-related harm among veterans, there remains a marked paucity of qualitative research capturing the lived experience of gambling harm in this population. Little is known about how veterans interpret their gambling behaviours, navigate stigma and self-concealment, or perceive the availability and appropriateness of support services. Moreover, there is limited insight into the nuanced pathways through which gambling problems emerge and escalate over time, or the psychosocial and institutional predictors that influence both risk and resilience. This study addresses these critical gaps by exploring the lived experiences of UK Armed Forces veterans affected by gambling-related harm. Through in-depth qualitative interviews, we examine how gambling behaviours develop and evolve, how military and post-military contexts shape gambling trajectories, and how individuals engage with or struggle to access support. The findings aim to inform the development of culturally sensitive prevention and intervention strategies that reflect the complexity and specificity of veterans’ lived experiences.

## Methods

### Theoretical underpinnings

We adopted a descriptive qualitative approach to guide this study, which is particularly well-suited to exploring how individuals experience and interpret specific phenomena.^25^ This method is particularly valuable for capturing rich, detailed accounts of participants’ views, especially in relation to emotionally sensitive or complex topics. A key strength of this approach is its emphasis on preserving participants’ language and meanings, while minimising abstract interpretation or theoretical imposition.^26^ Its clarity and accessibility make it especially appropriate for producing findings that are meaningful to both affected individuals and the practitioners who support them.^27^ This methodological choice was informed by an interpretivist epistemological stance, which recognises that knowledge is socially constructed and context-dependent.^28^ From this perspective, participants’ accounts are understood as situated within specific cultural and institutional contexts. The descriptive approach allowed us to stay close to participants’ narratives while remaining attentive to the subjective and socially mediated nature of their experiences.^29^ This was particularly relevant given the stigma and complexity surrounding gambling-related harm among veterans. The method’s flexibility also supported open-ended, participant-led interviews, which encouraged the emergence of unanticipated themes and enriched the depth of the data.^30^

### Sample and recruitment

UK former HM Armed Forces personnel were purposively recruited from a larger cross-sectional survey of veterans, as well as through veteran-related charities, support organisations and a targeted Facebook campaign aimed at veteran communities. This multipronged recruitment strategy was employed to ensure access to a diverse range of experiences and backgrounds. Eligibility criteria required participants to have previously served in the Armed Forces and to have to have self-identified as having experienced gambling-related harm either during or following their military service. All participants were required to have been discharged from active military service in 2010 or later and to be residing in the UK at the time of the interviews. This cut-off was selected to enable participants to provide clearer and more relevant reflections on how their gambling experiences were shaped by, or related to, their time in military service. Efforts were made to include veterans from varied military branches, ranks, age groups and lengths of service to capture a broad spectrum of perspectives. Recruitment materials clearly outlined the study’s focus on gambling harm to facilitate informed participation. The final sample size of fourteen was determined based on the principles of information power,^31^ with sufficient richness and variation in the data to support meaningful analysis in relation to the study’s aims. We also monitored for data saturation during coding, and no new codes or concepts emerged from the final interviews, indicating that saturation had been reached.^32 33^

### Data collection

Prior to formal data collection, a pilot stage was conducted with three veterans from our lived experience panel. This process informed several refinements to the interview schedule, including adjustments to language, tone and the ordering of questions to ensure greater clarity, accessibility and sensitivity. The pilot did not generate pre-defined analytic categories but was used to refine the phrasing and flow of questions to support open, participant-led discussion. It also confirmed the relevance of key topic areas and helped establish a trauma-informed approach suited to discussing potentially distressing material. A trauma-informed approach was operationalised throughout data collection by prioritising participant safety, choice and control. Participants were reminded that they could pause, decline to answer questions or stop the interview at any point without providing a reason. Interview pacing was guided by participants, with sensitive topics approached flexibly rather than in a fixed sequence. The interviewer remained attentive to signs of distress and checked in with participants when discussing potentially triggering material. All participants were debriefed at the end of the interview and provided with signposting to relevant support services.

14 one-to-one semistructured interviews were conducted between September 2024 and May 2025. All participants who consented to take part completed the interview. Each interview lasted approximately 45 to 60 min and was conducted via confidential Zoom meetings. Each participant took part in a single interview; no repeat interviews were conducted. Interviews followed a semistructured format to allow for flexibility while eliciting rich, in-depth data.^30^ The interview schedule was intentionally broad and non-directive, enabling participants to shape the focus and depth of discussion and allowing unanticipated issues to emerge. Open-ended questions were used to explore participants’ lived experiences with gambling harm, including their gambling history, pathways into gambling, motivating factors, control strategies, help-seeking behaviours during and after military service, and the impact of online gambling (interview schedule, online supplemental file 1).

Prior to the interviews, all participants were provided with an information sheet outlining the background and aims of the study. They were given time to review the materials, ask questions and provide written informed consent. On consent, interviews were audio-recorded and later transcribed verbatim. Transcripts were not returned to participants for comment or correction, in order to minimise participant burden and maintain confidentiality. All interviews were conducted individually by a trained member of the research team (DD), who had no prior relationship with the participants. Participants were compensated for their time with a £20 voucher. Due to the small and potentially identifiable nature of the sample, direct identifiers such as names, sex or age have been omitted. To support researcher well-being, the interviewer had access to regular opportunities for informal debriefing within the research team following interviews that involved potentially distressing material.

### Patient and public involvement

Patients and the public were involved in the early stages of the research through a lived experience panel of veterans, who contributed to piloting and refining the interview guide to improve clarity, sensitivity and relevance. They were not involved in recruitment, data collection, analysis or interpretation of the findings. Participants were not involved in the dissemination of the results.

### Data analysis

The interview data were analysed using reflexive thematic analysis as outlined by Braun and Clarke,^34^ and further elaborated in their reflexive approach^35^ following an inductive, data-driven approach.^36^ The reflexive orientation acknowledged the active role of the researcher in interpreting and constructing meaning from the data, while the inductive approach allowed patterns and themes to emerge directly from the data rather than being imposed by existing theoretical frameworks. This was essential given the exploratory nature of the research and the diversity within the sample. Reflexive thematic analysis was particularly suitable for this study, as it facilitated a nuanced understanding of varied perspectives without assuming a single explanatory theory.^37^ The Consolidated criteria for Reporting Qualitative research checklist was used to guide transparent reporting across study design, data collection, analysis and reflexivity, with relevant items addressed throughout the Methods section.^38^

Analysis followed the phases outlined by Braun and Clarke, including familiarisation with the data, inductive coding, iterative development of candidate themes, review and refinement of themes, and reflexive interpretation throughout the analytic process.

To maintain confidentiality, each participant was assigned a unique numerical code, and all identifying details were removed or anonymised during transcription. Transcripts were reviewed carefully to ensure anonymity and completeness before analysis. Initial coding was led by the lead researcher (DD) and was inductive in orientation, with codes generated through close engagement with the data rather than through the application of predefined categories. Existing literature informed the researcher’s analytic sensitivity but did not constitute a deductive coding framework.^36^ As analysis progressed, initial codes were grouped into broader themes through iterative comparison and reflection. To enhance the trustworthiness of the analysis, and in accordance with the criteria outlined by Elo,^39^ several measures were taken to ensure methodological rigour. Credibility was strengthened through deep immersion in the data, involving multiple close readings of transcripts, line-by-line coding and the use of ongoing memo-writing. Reflexivity was embedded throughout the analytic process, with the lead researcher engaging in ongoing reflection on how their assumptions, positionality and prior engagement with the topic shaped interpretation. Collaborative input from members of the research team further supported the dependability and confirmability of the findings. Consistent with a reflexive thematic analysis approach, team discussions were used to support reflexive dialogue and analytic refinement rather than to achieve consensus coding or inter-rater reliability. Themes were reviewed and refined through regular team discussions, ensuring clarity, coherence and alignment with the study’s aims.

NVivo (V.22) software was used to support the organisation of transcripts, systematic coding and memo development throughout the analytic process. Iterative comparison across transcripts was used to explore similarities and differences within the dataset; references to constant comparison draw on general analytic principles rather than indicating adherence to grounded theory as a methodological framework.^40^ To ensure that the findings remained grounded in participants’ lived experiences, the results are illustrated with rich, representative quotations.

## Results

### Study participants

We recruited fourteen UK Armed Forces veterans, all of whom reported a personal history of gambling-related harm. A summary of participants’ demographic and military service characteristics is presented in table 1.

**Table 1:** Participants’ characteristics

Sample characteristics	Participants N (% of total)
Total	**N=14**
Sex	
Female	n=1 (7%)
Male	n=13 (93%)
Age (years)	
25–34	n=2 (14%)
35–44	n=3 (21%)
45–54	n=5 (36%)
55–64	n=4 (29%)
Military branch	
Army	n=4 (29%)
Navy	n=7 (50%)
Royal Air Force	n=3 (21%)

### History of gambling harm

All participants reported experiencing gambling-related harm, with many describing a gradual progression from casual gambling, often initiated during their military service, to behaviours they later recognised as compulsive and unmanageable, frequently concealed from others.

#### Reflexive thematic analysis

Reflexive thematic analysis generated four major themes through an iterative analytic process involving inductive coding, development of candidate themes and ongoing refinement through reflexive interpretation. Four major themes were identified: (1) cultural normalisation of gambling in UK and military contexts; (2) gambling preferences and motivations during military service; (3) barriers and supports for help-seeking during service and (4) escalation of gambling post-military life and its social impact. The themes and subthemes are summarised in table 2 and illustrated with anonymised participant quotes throughout the Results section. Additional illustrative quotations that support the themes but are not reproduced in full in the main text are provided in online supplemental file 2.

**Table 2:** Summary of themes and subthemes that arose from the interviews

Overarching theme	Subtheme	Description
Cultural normalisation of gambling in UK and military contexts	Gambling as a normalised part of civilian life	Describes gambling as a familiar and accepted activity from an early age, often introduced within families, schools and local communities.
	Gambling as a normalised part of military settings	Describes how gambling was embedded in military life, reinforced by boredom, group bonding and institutional routines.
Gambling preferences and motivations during military service	Gambling preferences and motivations	Describes participants’ preferred types of gambling activities and the motivation behind their choices.
	Evolution in the nature and intensity of gambling	Describes how their gambling escalated over time, often shifting from casual or social forms to more compulsive and high-risk behaviours.
Barriers and supports for help-seeking during service	The dichotomy of military support	Describes conflicting experience where formal support services existed, yet a military culture of stigma, shame and fear of negative consequences created a barrier to accessing help.
	Internalised stigma and the cycle of financial harm	Describes how internalised stigma, shame and fear of being judged contributed to the concealment of their gambling behaviours. This secrecy often led to increased emotional isolation, reluctance to seek support and a worsening of both psychological distress and financial consequences.
Escalation of gambling post-military life and its social impact	Civilian Transition and Escalation of Gambling	Loss of military structure, identity and connection after discharge contributed to increased gambling. Participants described boredom, emotional distress and the ease of online access as driving factors.
	The Impact of Gambling on Family and Social Relationships	Participants described how escalating harm strained relationships, eroded trust and led to isolation from partners, family and friends.

#### Theme 1: cultural normalisation of gambling in UK and military contexts

Participants described how gambling was embedded in both civilian and military environments, shaping their early exposure and later habits. This theme captures how social and institutional cultures contributed to the perception of gambling as a normative, low-risk activity, often masking the development of harm.

#### Subtheme 1.1: gambling as a normalised part of civilian life

For many participants, gambling was introduced early in life as a routine and socially accepted activity. A particularly common reference was the Grand National (an annual UK horse racing event), which several participants recalled as a family tradition. This event often marked their first experience of betting and was remembered as a fun, communal activity shared with parents and siblings:

You’ve got the Derby. You’ve got the Grand National there, you know. Really, you got loads of races, horse races all over the country all the time (P08-RAF) I was always really disruptive on family holidays, but one year I found this machine in the arcade, the penny pushers, and I sat there for hours. My mother was happy I was kept occupied for once. (P07-Navy) (Referring to arcade coin-operated machines that can be used by children, in which coins are dropped onto a moving shelf to push other coins off the edge).

Several participants noted that while they received education in school about the risks of smoking, drugs or alcohol, gambling was not mentioned at all. This absence of early warnings reinforced a view that gambling was a harmless form of entertainment.

We got loads of talks about drink and drugs at school. Gambling? Nothing. No one ever mentioned it. (P12-Army)

Gambling machines were described as being deeply embedded in everyday civilian environments, making them difficult to avoid. Participants spoke of their visibility in pubs, service stations and other social venues, where their constant presence made casual gambling feel routine and almost unavoidable:

Just on the High Street. Any pub you walk into. There’s 3 or 4 [machines], you know. Go to Wetherspoons, there’s 6 or 7 of them. They’re just wherever you go. You can’t get away from them (P14-Navy). (‘High Street’ refers to a town’s main commercial street; ‘Wetherspoons’ is a large UK pub chain).

Together, these early experiences shaped a cultural context in which gambling was socially reinforced and seldom viewed as problematic. This sense of normality shaped later attitudes and made it more difficult for individuals to recognise harmful patterns as they developed over time.

#### Subtheme 1.2: gambling as a normalised part of military settings

Participants consistently described how gambling was embedded into the rhythms of daily life during military service. Gambling activities were often accessible, encouraged or even institutionalised through ‘mess halls’, deployment routines and social events. Rather than being seen as a harmful behaviour, gambling was viewed by many as a sanctioned way to alleviate boredom, bond with peers or decompress after intense operational periods.

Some participants reflected on how gambling became a regular part of life during downtime:

It was just part of the routine. You’d go to the NAAFI, grab a drink, have a flutter on the fruit machines. Everyone did it, so you didn’t question it. (P02-Navy). ‘NAAFI’ refers to the UK Armed Forces retail and leisure organisation operating on military bases; ‘fruit machines’ are UK slot machines commonly found in pubs and clubs).

Others noted that gambling was often promoted as a group activity during social or fundraising events, making it feel not only acceptable but positively reinforced:

We had race nights and raffles all the time. You’d be encouraged to get involved. It was for morale, for charity (P06-RAF)

Two different methods of normalisation. Really, when I was away with the Navy we’d gamble in casino environments. If we was away at sea, we used to hold horse racing nights where you would actually place your bets with the captain of the ship. (P11-Navy)

In some cases, participants suggested that gambling served as a means of maintaining inclusion within the military’s social fabric. Declining to participate in group gambling activities could carry implicit social penalties:

You’d be outcasted. Or you’d be like, something’s wrong with you… In some cases, I would even think that it would affect promotions and things because you’re not social. You don’t know your bosses. (P04-RAF)

It’s not just socially amongst your peers. It could be with higher ups as well. Then that would be an opportunity to get close to them (P13-Army)

The availability of gambling machines on bases, and the lack of restrictions or warnings around use, created an environment where potentially harmful behaviours could develop unnoticed:

The machines were right there in the corner of the bar. You’d stick a tenner (£10) in just to pass the time. I didn’t realise how much I was using them until I was out. (P14-Navy)

For some, the culture also masked early signs of harm. Gambling was normalised to such an extent that excessive use was rarely challenged:

I lost loads on tour, just kept going back to the machines. But no one really said anything. It was just something we all did. (P03-Navy)

I thought, well, I’ve met normal people. These people are in the services and they’re gambling. This is normal. I’ve been telling people for years what I’m doing is normal. (P07-Navy)

This permissive environment, combined with military norms of self-reliance and resilience, meant that gambling behaviour was rarely discussed openly, even when it became problematic. Several participants reflected that the culture encouraged them to internalise and hide early signs of distress:

You’re not going to put your hand up and say you’ve got a problem with gambling. That’s just not how it works. You deal with it yourself. (P05-Navy)

These accounts highlight how military environments can foster the development of gambling behaviours by offering both opportunity and perceived acceptability, while simultaneously discouraging open dialogue about harm.

#### Theme 2: gambling preferences and motivations during military service

Participants provided detailed accounts of the types of gambling they engaged in while serving, and their motivations for gambling during this period. This theme explores not only the kinds of gambling that were popular but also the psychological and situational drivers behind these behaviours.

#### Subtheme 2.1: gambling preferences and motivations

Participants frequently described a clear preference for non-skilled, chance-based gambling activities such as fruit machines, fixed-odds betting terminals (FOBTs) and roulette. These activities were often chosen not for entertainment or financial gain, but as a means to mentally disconnect, self-soothe or temporarily escape from emotional strain and stressors linked to military life.

This preference for passive, repetitive gambling formats was commonly linked to their ability to help participants ‘switch off’ or achieve a dissociative state:

It was more the machines than anything else. I didn’t want to think. I didn’t want to concentrate on poker or anything like that. I just wanted something that I could do and completely zone out. (P05-Navy)

I used to get more highs off roulette because it was out of my control, and it was like luck and sports betting if I watch a game and I bet on the 1st goals for it. It was out of my control. And I get more adrenaline highs if they won. (P08-RAF)

For many, the appeal lay in the mechanical repetition and predictability of these games, which contrasted with the high-pressure and unpredictable nature of military work. In this sense, gambling functioned less as a competitive or strategic exercise and more as an emotional coping tool:

People assume it’s for excitement or money. But for me, it was the opposite. It was about switching everything off. The flashing lights, the sounds—they helped block things out. (P07-Navy)

These accounts reveal that participants’ gambling choices were shaped by the mechanics of the games insofar as these mechanical features provided a vehicle for temporary emotional relief. The simplicity and accessibility of fruit machines and roulette games made them ideal tools for escapism in a military context where direct expressions of vulnerability were often discouraged.

#### Subtheme 2.2: evolution in the nature and intensity of gambling

Many participants described a clear progression in their gambling behaviour during military service. What began as casual or social engagement often intensified into high-frequency, high-stakes gambling. This escalation was driven by a combination of boredom, stress relief, emotional numbing and a perception that gambling was a low-consequence activity within the structured environment of military life.

Some participants explained how the nature of their gambling changed as they became more emotionally or financially invested over time:

At the start it was just something to do, a bit of a laugh with the lads. But before I knew it, I was spending hundreds a week, chasing losses, going back even when I was skint. (P06-RAF)

It changed over time. First it was a few quid (a few British pounds) here or there, but then it was all the time. It got to a point where I needed it, like, I couldn’t relax unless I gambled. (P03-Navy)

I won two and a half grand off, maybe a hundred quid or something. So that was a key moment, because that was right near the beginning, so that like lifted me as well, and then it was like a lifestyle (P04-RAF)

The structure of military life often meant that participants had few living costs, with accommodation and meals provided, reducing the immediate impact of financial loss. Several described how this environment made gambling feel less risky:

I did have big wins, and then it felt like pocket money, like RAF, like military money is just pocket money, roof over my head, and I knew I would have my meals paid for. (P04-RAF)

That money was not for bills, or for holidays, or for anything else. That was my gambling tokens. And the only way I relate to it is, you know… all of that cash was like ten bags of heroin to a heroin addict. You’re not going to pay your bills with that heroin. (P07-Navy)

This detachment allowed participants to minimise consequences and continue gambling. Over time, gambling became more compulsive and less social, with individuals increasingly using it to escape negative emotions or mental distress:

It got more and more serious. I’d go in on my own, just to zone out. I didn’t even care if I lost, I just didn’t want to think. (P13-Army)

These accounts demonstrate how gambling shifted from a seemingly harmless pastime into a compulsive cycle, driven by emotional regulation needs, enabled by military living conditions, and sustained by avoidance of financial and psychological consequences. Over time, this escalation often led to secrecy, debt and feelings of helplessness, even while still in service.

#### Theme 3: barriers and supports for help-seeking during service

Participants shared complex experiences around seeking help for gambling-related harm during military service. While some were aware of available support systems, few accessed them. The prevailing military culture of discipline, stoicism and performance made many reluctant to disclose gambling problems. Participants described a pervasive culture of stigma, fear and silence that discouraged open conversations about harm, even when formal support structures technically existed.

#### Subtheme 3.1: the dichotomy of military support

Participants offered conflicting accounts of the support available to them during service, revealing a complex and often contradictory relationship between formal support structures and informal cultural barriers. While some recalled being encouraged to speak to welfare staff, chaplains or chain of command about personal difficulties, others described these pathways as ineffective or even risky to engage with:

We’d always been encouraged since joining up, if you’ve any worries or anything, talk to a captain and they’ll help you. I didn’t get any help. (P11-Navy)

In several cases, participants who did reach out for help encountered direct or implied threats of disciplinary or career consequences. Seeking support for financial difficulty or gambling harm was frequently met with caution or deterrence, reinforcing a message that vulnerability could result in punitive outcomes:

During the military I knew I needed help. I asked for help, only to be told, mate, if I ask for this help for you, you’re going to be discharged for financial responsibility. (P06-RAF)

I was basically told, look, if you start saying you’ve got money problems and gambling problems, that’s going to open a whole can of worms. They’ll look at whether you’re fit to serve, and if you can’t manage your finances, that’s grounds for discharge. (P10-Army)

This climate of fear was intensified by accounts of severe institutional repercussions. Only one participant described formally disclosing their gambling-related financial distress while still in service. Rather than receiving support, the consequence was punitive:

I ended up in the MCTC for 13 months, and I wasn’t getting paid during that time either. All because I told them I was in debt and needed help. (P13-Army)

At the Military Corrective Training Centre, he described a prolonged struggle to access support services, reinforcing how difficult it could be to navigate the system even after coming forward:

When I was at MCTC, I had to push and push to get any kind of help. Eventually they brought in someone from SSAFA, and I started getting proper advice. But it didn’t come easy. (P13-Army). (“SSAFA” refers to a UK Armed Forces charity providing welfare and financial support to service personnel and veterans).

This experience illustrates how formal help-seeking could result in disciplinary action rather than care, strengthening the perception that disclosing financial vulnerability was not only stigmatised, but also professionally dangerous and personally costly. It also underscores how the institutional framework often placed the burden on individuals to advocate persistently for support, even in punitive or high-stress settings, and how access to meaningful help was inconsistent and far from automatic, even when the need was clear.

Consequently, many chose not to disclose their difficulties at all, fearing it would jeopardise their standing, relationships or future in the forces. Others internalised their struggles and avoided official support systems altogether:

I knew I was struggling, but admitting it felt like failure. In the Forces, you’re supposed to crack on, not complain. (P10-Army)

This fear of negative consequences created a powerful disincentive for disclosure, even among those who recognised they were struggling. Participants also highlighted inconsistencies in how help was offered, with some citing instances where peers were supported, while others were reprimanded or marginalised for seeking assistance:

It all depends who you speak to. One lad got counselling, but another got demoted. There’s no consistency, so people just keep quiet. (P09-Navy)

These findings reveal a disconnect between the apparent availability of support and the cultural context that often prevented its effective use. Although formal mechanisms existed, they were frequently inaccessible in practice due to the pervasive stigma, fear of reprisal and inconsistent institutional attitudes that shaped the everyday experiences of personnel navigating gambling harm.

#### Subtheme 3.2: internalised stigma and the cycle of financial harm

Participants described how internalised stigma, shame and fear of being judged contributed to the concealment of their gambling behaviours. This secrecy often led to increased emotional isolation, reluctance to seek support and a worsening of both psychological distress and financial consequences. Several participants expressed that acknowledging a problem with gambling would have been perceived as a personal weakness or a failure to cope:

The military taught me not to show my emotions when I was in training…No, you don’t show you want to show your emotions because it’s a weakness. (P01-Army)

The pressure to maintain a stoic or self-reliant persona meant that gambling often became an unspoken coping mechanism. For some, concealing their gambling was tied to the fear of disciplinary or reputational damage, while for others, it was linked to personal shame or a desire to maintain control in the eyes of their peers and family:

I never told anyone. It was easier to act like everything was fine. I couldn’t admit I was losing control. (P06-RAF)

You’re always supposed to be sorted, squared away. Saying you’re in debt or struggling would make people think you can’t handle yourself. (P10-Army)

Several participants described how financial losses due to gambling not only caused significant hardship but also reinforced continued gambling behaviour. This formed a self-perpetuating cycle, where attempts to recoup losses through further gambling were driven by desperation, stigma and the absence of alternative coping mechanisms:

I didn’t want to admit it, so I kept betting, thinking I’d win it back before anyone found out. But the hole just got deeper. (P12-Army)

You’re already in debt, so stopping feels like accepting failure. Carrying on feels like the only way. (P05-Navy)

As gambling intensified, several participants began to borrow money, often through payday loans or from family members, to cover losses or continue gambling. This escalation was often done in secrecy, further entrenching feelings of shame and isolation:

I ended up getting payday loans, one after the other. I wasn’t thinking straight, just trying to win it back. (P05-Navy)

I had to borrow off my brother a few times. Told him it was for rent or food. I couldn’t tell him it was because I’d lost everything on roulette. (P02-Navy)

In some cases, participants described making significant sacrifices or taking extreme measures to cover up the extent of their financial distress. These efforts to maintain a façade of control further delayed help-seeking and deepened the emotional toll of gambling harm:

I was lying to my wife constantly. Saying money went on bills or car stuff. I was terrified she’d find out how much I’d lost. (P03-Navy)

For a number of participants, the cumulative impact of unmanageable debt, secrecy and self-blame led to severe emotional crises. A small number openly disclosed suicidal ideation, directly linking their mental health struggles to gambling harm and the isolating nature of stigma:

I had plans in place to take my own life. I’d written the notes and everything. That’s how bad it got because I couldn’t see a way out. (P12-Army)

Chose to end my life than open my mouth and ask for help, then deal with the shame and stigma and embarrassment that I thought would come with that. (P11-Navy)

These accounts underscore the profound psychological consequences that can emerge when shame and silence prevent early intervention. The fear of being judged or penalised led many participants to persist in secrecy, even as their circumstances became more difficult to manage.

Together, these accounts illustrate how the internalisation of shame and stigma not only inhibited disclosure and access to support but also contributed to a cycle of financial harm that became harder to escape. In the most severe cases, the combination of emotional isolation and financial collapse led participants to contemplate suicide, highlighting the urgent need for more responsive, non-punitive and stigma-free support systems.

#### Theme 4: escalation of gambling post-military life and its social impact

For many participants, gambling did not stop after leaving the Armed Forces; instead, it often escalated. While the military environment had introduced or reinforced gambling behaviours, it was the transition to civilian life that intensified their impact. Participants described how the sudden absence of military routine, structure and peer support left them vulnerable to emotional distress, boredom and social isolation. Gambling began to serve not just as entertainment, but as a coping strategy for navigating the complexities of postservice life. Financial pressures, easier access to online platforms and difficulties adjusting to civilian roles all contributed to continued or intensified gambling. For some, this escalation also led to significant harm in personal relationships, including broken trust, separation from partners or children and deepened feelings of shame and isolation.

#### Subtheme 4.1: civilian transition and escalation of gambling

While some participants had encountered gambling prior to joining the Armed Forces, it was during service that gambling became more routine. After discharge, however, the behaviour often intensified. The transition to civilian life brought a loss of structure, identity and camaraderie, creating a void that gambling sometimes filled. In this context, gambling became more frequent, accessible and emotionally charged:

You have a lot more time to think, a lot more time to actually find something else to do, and feeling lonely, then you’re concentrating on something else. Your mind isn’t going a hundred miles an hour on the same subject. (P08-RAF)

I guess the escalation in my gambling came when I found out that I would be leaving the forces, and it came alongside excessive consumption of alcohol. (P11-Navy)

Veterans also reflected on financial changes after discharge. Although they now faced new responsibilities such as rent, bills and household expenses, they were unaccustomed to managing money in this way. During service, accommodation and food were provided, and salaries were perceived as disposable income. This shift left some struggling to budget and more vulnerable to using gambling as a financial coping strategy or a familiar outlet for stress:

I think I got a resettlement grant of about 3.5 thousand pounds that you could spend on a civilian course. But I just saw it as money to burn, to be honest. I wasn’t used to paying bills or budgeting. In the military, you didn’t think about that stuff. (P01-Army)

Alongside financial instability and boredom, participants described how technology increased the availability and privacy of gambling. While military gambling often took place in physical venues, post-service life introduced new risks through the availability of online gambling. For some, constant online access contributed to continued harm. The ability to gamble privately at any time and from any location removed external barriers and made it easier to hide problematic behaviours:

Well, it became a lot easier if you didn’t have to go out and be anywhere, and you could just be home and switch on your laptop, and it’d be there in front of you. So it became a lot easier to do. (P11-Navy)

You could just do it without going anywhere. Just sit at home on your phone and lose money without anyone knowing. (P08-RAF)

Efforts to control gambling often failed, despite growing concern. Some participants described how even attempts to self-regulate their gambling, such as self-exclusion, often failed over time:

*I* then banned myself, a two year ban, thinking that’d do it, could deal with that, but then I came back after the ban ended, within about six months I was back doing it again. (P14-Navy)

I tried setting limits and timers on the app, but it was easy to ignore. You just click a few buttons and carry on. (P09-Navy)

These accounts illustrate how the postservice period often intensified gambling-related harm, shaped by the emotional and structural void left after discharge. For many, gambling became a way to manage the difficult shift out of military life. While some recognised their struggles and attempted to exert control, these efforts were often undermined by the same psychological and environmental pressures that drove them to gamble in the first place.

#### Subtheme 4.2: the Impact of gambling on family and social relationships

As gambling intensified after military service, its consequences became deeply entangled with participants’ personal lives. Many described the toll that secrecy, debt and emotional withdrawal had on their relationships with partners, children and close friends. While gambling had initially served as a coping mechanism, its long-term impact often included isolation, mistrust and broken bonds.

Participants frequently spoke about the damage caused by dishonesty and concealment. Efforts to hide gambling behaviour or the scale of associated debt led to repeated breaches of trust within families:

My family didn’t know how bad it was. I was lying constantly. Telling them I was skint because of bills, but really, I’d blown it all on roulette. (P09-Navy)

I used to sneak out after dinner, tell my wife I was going for a walk, and head to the bookies. Every time I came back, I felt sick with guilt. (P10-Navy)

For some, the consequences were relational collapse. Discoveries of debt, deceit and compulsive behaviour resulted in estrangement or the end of intimate partnerships:

My wife would threaten to kick me out if I gambled again. And I promised her I wouldn’t. But I always did. It got to the point where she just stopped believing anything I said. (P07-Navy)

I lost my kids for a bit. My ex took them and said I wasn’t fit to be around them when I was gambling. That was my rock bottom. (P05-Navy)

Others described how gambling affected the emotional climate of their home life, eroding communication and increasing tension. These dynamics contributed to distance and conflict, even in relationships that remained intact:

I was horrible to my missus when I gambled. I wasn’t violent, but I was just short, irritable, snapping all the time. She stopped wanting to talk to me. (P03-Navy)

My kids started noticing something was wrong. I wasn’t really there. I’d sit with them but be thinking about getting online again. (P06-RAF)

Social networks also became strained. Friends began to withdraw, and participants found themselves isolated, sometimes as a result of manipulative behaviours driven by gambling needs:

My mates stopped inviting me out. I’d always try to borrow money or disappear halfway through the night to gamble. They had enough. (P06-RAF)

Taken together, these accounts highlight how gambling-related harm extended beyond the individual to affect those closest to them. These relational costs, formed as guilt, secrecy and disconnection, intensified shame and further hindered support-seeking. For many, gambling not only replaced the routine and camaraderie of military life, but also progressively eroded the social anchors that remained.

## Discussion

This study explored the lived experiences of gambling-related harm among UK Armed Forces veterans, with a focus on how military culture, psychological factors and institutional structures shaped gambling trajectories, barriers to help-seeking and long-term social impacts. Reflexive thematic analysis generated four interrelated themes, each of which reflects the complex interplay between cultural normalisation, psychological vulnerability, structural stigma and life transitions. These findings build on and extend current understanding of gambling harm in military contexts, offering critical insights into the social processes that render such harm both pervasive and hidden. Consistent with a reflexive thematic analysis approach,^35 41^ the themes presented do not claim to represent objective truths or universal mechanisms, but rather reflect the researchers’ interpretive engagement with participants’ situated accounts. Existing theoretical frameworks are used in the Discussion as sensitising concepts to deepen interpretation, rather than as explanatory models imposed during analysis. This positioning aligns with the epistemological foundations of reflexive thematic analysis, which emphasise reflexivity, context and meaning making over prediction or theory verification.

### Military and civilian cultures as sites of gambling normalisation

Gambling was described as embedded across the life course of the UK culture, from early exposure in civilian contexts to routine engagement during military service. This reflects findings on gambling’s cultural normalisation in both society^1 42 43^ and within military settings.^11 24^ Participants portrayed gambling during service as widespread and institutionally tolerated, driven by boredom, downtime routines and peer influence.

The social acceptability of gambling was reinforced by group cohesion and a lack of scrutiny, with behaviours framed as morale-building rather than risky. These align with research showing that military environments often position gambling as ordinary or even beneficial,^3 11 44^ obscuring harm and complicating help-seeking.^45 46^ Within rigid hierarchies and cultures of emotional control, gambling may become normalised, concealed and difficult to detect, particularly when embedded in everyday institutional practices.^1 13^

### Psychological coping and escalation during service

Participants described using fast-paced, non-strategic gambling as a way to detach from stress, emotional strain and cognitive overload during military service. Rather than being motivated by financial gain or excitement, gambling functioned as a means of switching off, managing mood and achieving emotional distance during downtime or after demanding shifts. This pattern aligns with research on avoidance-based emotion regulation in institutional settings characterised by high performance demands, restricted autonomy and norms of emotional control.^7 20 47^

While trauma-related explanations may offer one relevant lens,^16 46^ participants’ accounts in this study point to a broader affective process. Gambling was actively selected for its capacity to induce immersion, time-loss and dissociative states, particularly through electronic gambling machines and FOBTs, which have been shown to facilitate these effects.^48^,^50^ In this context, gambling functioned as an avoidant coping strategy, consistent with the escape/relief pathway described by Blaszczynski and Nower,^51^ providing temporary relief from distress rather than entertainment or reward.

Participants also described a gradual and often unnoticed shift from casual to compulsive gambling. This escalation was facilitated by steady income, limited immediate financial consequences during service and a cultural emphasis on self-reliance and stoicism that discouraged disclosure or help-seeking. Similar structural enablers have been identified in serving personnel.^24^ However, participants’ narratives in the present study illustrate how these conditions interacted to normalise gambling as a privately damaging but socially acceptable outlet for distress, allowing harm to develop largely unchecked. This extends existing evidence by illuminating the lived mechanisms through which cultural norms and institutional structures sustain gambling escalation during service.^52^,^55^ This analysis clarifies how gambling becomes normalised as a functional coping strategy within military life, rather than merely a co-occurring behaviour.

### Stigma, silence and structural barriers to help-seeking

Stigma emerged as a key barrier to disclosure. Participants described withholding help-seeking due to fear of reputational damage, disciplinary action or forced discharge. While some recalled nominal encouragement to seek support through welfare services or chaplaincy, these pathways were often perceived as inconsistent, inaccessible or actively risky. The coexistence of formal support structures and informal sanctions created a climate of mistrust and ambiguity.

These findings align with existing research on stigma and support in military contexts^19 55^ and highlight how gambling is frequently overlooked or mishandled. Rather than initiating care, disclosure could trigger punitive responses. This contradiction reflects a form of structural stigma, where institutional messaging about support is undermined by its consequences.^52 54 55^ In this context, gambling harm was not only stigmatised but also framed as a professional liability. This insight adds nuance to debates on military mental health support by showing that barriers to care arise not from the absence of support, but from the risks attached to using it.

Internalised stigma intensified these challenges. Participants described feeling pressured to appear ‘sorted’ and emotionally self-sufficient, echoing military norms of discipline, control and resilience.^52 54^ Such norms are consistent with evidence on avoidant coping in military personnel, where emotional suppression and hyper-independence may delay intervention.^53^ Several participants reported significant emotional deterioration, including suicidal ideation, illustrating how stigma can escalate distress and deepen harm. Given the normalisation of gambling within military life, systems must be prepared for addiction-related harm. Support responses need to be consistent, non-punitive and culturally responsive. Without this, gambling-related distress is likely to remain concealed and unaddressed.

### Postservice life as a locus of escalation and isolation

The post-military transition emerged as a critical period during which gambling often escalated. The loss of military routine, identity and structure left many participants vulnerable to boredom, emotional strain and isolation. Consistent with Fry *et al*^5^ and Adams *et al*,^15^ gambling became more frequent, immersive and emotionally charged after discharge, particularly as participants sought ways to manage unstructured time and loneliness. The accessibility of online gambling further intensified this shift, enabling private, continuous engagement without external scrutiny.^1 56^

While transition has been widely recognised as a period of vulnerability, this study sheds light on the lived processes through which gambling becomes embedded during this period. Participants described using gambling to manage boredom, identity loss and emotional dysregulation following discharge. Their accounts indicate that transition often marked a turning point that preceded deeper escalation, including worsening debt, relationship breakdown, social isolation and severe psychological distress.

Financial mismanagement compounded this escalation. Many participants lacked experience managing civilian expenses and described using gambling as a financial escape, often drawing on discharge payments or credit in secrecy. The relational consequences were substantial, with participants reporting relationship breakdown, loss of contact with children and social withdrawal, alongside suicidal ideation.^57 58^ Attempts at self-regulation, such as self-exclusion or betting limits, were commonly ineffective, aligning with evidence that individual control strategies often fail without systemic support.^59^

### Strengths, limitations and implications

This study offers rich, first-person accounts of gambling harm in a group underrepresented in the literature. By centring the voices of veterans, it provides insight into not only the risk factors but also the social and institutional contexts that shape harm trajectories. A key strength lies in its qualitative design, which allowed for in-depth exploration of complex and sensitive themes that are often overlooked in survey-based research. Nonetheless, reflexive thematic analysis is not without limitations. Its flexibility, while advantageous for exploring subjective experiences, may also introduce the possibility of researcher influence.^60^ Although inductive coding aims to reduce bias, a researcher’s background and assumptions can still shape how themes are constructed.^61^ However, rather than being viewed solely as a source of bias, this subjectivity can be understood as a valuable analytic resource.^62^ The active role of the researcher in interpreting and constructing meaning is recognised as part of the analytic process.^41^ Collaborative discussion of codes and themes supported reflexive dialogue, helping to incorporate diverse perspectives and enhance analytic rigour.

This sample, while diverse in-service background, was small and self-selecting, potentially limiting generalisability. Although the sample size was guided by the concept of information power,^31^ data saturation was also observed during coding, with no new codes or concepts emerging in the final interviews.^32 33^ While some authors have proposed numerical minimum sample sizes for thematic analysis, such thresholds are not a requirement within reflexive thematic analysis, where analytic adequacy is determined by information power, analytic depth and reflexive engagement rather than participant counts.^35^ All participants had already identified themselves as having experienced gambling-related harm, which may not reflect those with subclinical or unacknowledged problems. Only one participant identified as female, limiting the ability to explore potential gendered differences in gambling experiences, help-seeking behaviour or institutional responses. Additionally, while the study included veterans from three service branches, intersectional perspectives (eg, related to ethnicity, gender or lesbian, gay, bisexual, transgender and queer (or questioning) (LGBTQ+) status) were limited and warrant further investigation.

Despite these limitations, the findings suggest several important directions for policy and practice. First, routine gambling screening should be introduced across the Armed Forces, including pre-enlistment, active service and discharge processes. Evidence-based tools tailored to military populations^22^ must be implemented consistently. Second, stigma reduction efforts need to be embedded not only in education and training, but also within disciplinary systems to ensure that help-seeking does not lead to professional penalty. Finally, tailored postdischarge interventions should address the unique psychological, financial and relational challenges veterans face, with a particular focus on transitions and identity loss. The findings also highlight the importance of interventions that explicitly address the links between gambling harm, emotional distress and suicidality. Several participants described suicidal ideation in the context of escalating debt, shame and isolation. Future interventions should therefore integrate gambling harm support with mental health and suicide prevention pathways. This includes training military and veteran-facing services to recognise gambling-related distress as a potential suicide risk factor, and ensuring clear, non-punitive referral routes to psychological and crisis support. Trauma-informed, confidential approaches that promote early disclosure may be critical in preventing escalation to crisis.

## Conclusions

This study provides a detailed account of how gambling harm is shaped by cultural, institutional and psychological factors within and beyond military service. Gambling is not merely an individual problem, but a socially constructed and institutionally sustained phenomenon that emerges from specific high-risk, occupational environments. Despite its prevalence and impact, gambling is not currently recognised as a priority issue within UK military or veteran health systems, resulting in a lack of screening, data collection and tailored support. To prevent harm, interventions must go beyond individual education and include systemic changes to how gambling is understood, addressed and supported across the Armed Forces and veteran care systems. Efforts to normalise disclosure, provide culturally competent care and disrupt harmful norms around self-reliance and emotional suppression are essential if we are to reduce gambling harm and its devastating consequences among UK veterans. By foregrounding veterans’ lived accounts, this study adds explanatory depth to existing prevalence research, illustrating how known risk factors translate into cumulative harm across military and post-military life.

## Supplementary material

10.1136/bmjopen-2025-109458 online supplemental file 1

10.1136/bmjopen-2025-109458 online supplemental file 2

## Data Availability

All data relevant to the study are included in the article or uploaded as supplementary information.
