# Supplementary material for: Qualitative exploration of gambling harm among UK veterans: normalisation, stigma and postservice escalation
Source: BMJ Open. 2026 Mar 25;16(3):e109458. doi: 10.1136/bmjopen-2025-109458 (PMC13034226; doi:10.1136/bmjopen-2025-109458)
Supplement: online supplemental file 1 [file bmjopen-16-3-s001.docx]

**Veterans HABIT Study - Semi-structured Interview Schedule**

1. **Introductory questions**
2. Can you tell me a little about yourself? (age, occupation, relationship status, military background and discharge date etc.)
3. What was the reason for your discharge?
4. Did you get any kind of support when you left the military? What was it, if any?
5. **Exploration of the core phenomenon**
6. Can you recall the time when you placed your first bet? Can you tell me about it? Do you have a preference to certain types of gambling? If so, which ones do you typically engage with?
7. Has your gambling preferences changed at all over time? If so, can you identify a cause for that change?
8. Would you say that gambling was linked in any way to your military service?
9. Did you feel it was normalized in the military?
10. Would you say you were gambling more or less during your service compared to being a civilian?
11. **Pathways to gambling behaviours and control measures**
12. Can you identify what motivates you to gamble?
13. Looking back, can you identify any events or incidents that would encourage you to decide to gamble?
14. Have you ever noticed anything that discouraged you from gambling?
15. Do you ever gamble with or around friends and family?
16. Is being around friends and family likely to encourage or discourage you from gambling?
17. Was there anyone in your life that affected your gambling behaviour in a negative or positive way? If so, how?
18. **Impact of advertising on gambling**
19. How often do you use free promotions (such as free bets, deposit bonuses, or sign-up) bonuses from gambling advertisements?
20. What do you think about these types of offers in gambling ads?
21. Did online gambling change your gambling preferences or frequency?
22. **Help seeking**
23. Did you ever feel like you needed support in managing your gambling activities? If so, did you reach out for help before?
24. What prompted you to seek support? Prompt: Did any event motivate you? Did someone else have a role to play in your decision?
25. What was your experience of seeking and receiving help like? Prompt: Did you get what you expected, or wanted, from seeking help? Were there any downsides?
26. What made you feel that your gambling is impacting your life or the lives of those close to you?
27. **Concluding comment**
28. What are the next steps for you in terms of your involvement in gambling?
29. Is there anything that was not asked today, and you feel will be important to add?
