# Supplementary material for: Qualitative exploration of gambling harm among UK veterans: normalisation, stigma and postservice escalation
Source: BMJ Open. 2026 Mar 25;16(3):e109458. doi: 10.1136/bmjopen-2025-109458 (PMC13034226; doi:10.1136/bmjopen-2025-109458)
Supplement: online supplemental file 2 [file bmjopen-16-3-s002.docx]

**Theme 1: Cultural Normalisation of Gambling in UK and Military Contexts**

**Sub-theme 1.1: Gambling as a Normalised Part of Civilian Life**

*"They drilled it into us about the dangers of smoking and drinking, even drugs—but gambling was never brought up. It just wasn’t on the radar, so you don’t think it’s a problem."* (P01-Army)

*“Everywhere you looked there were machines. In the pub, at the train station, even the bloody service stops.”* (P10-Army)

**Sub-theme 1.2: Gambling as a Normalised Part of Military Settings**

*“I didn’t think much of it at the time. Stick twenty quid in the machine, have a laugh. But it builds up. No one’s telling you to stop.”* (P14-Army)

*“We were supposed to be tough. You didn’t talk about it even if you were losing money. You’d just crack on.”* (P12-Army)

**Theme 2: Gambling Preferences and Motivations During Military Service**

**Sub-theme 2.1: Gambling Preferences and Motivations**

*“I would use roulette, because on that game you could win or lose so quickly. So roulette was always the one when I'd won a lot of money.”* (P09-Navy)

*“It wasn’t the thrill that kept me there, it was the silence in my head. Everything else goes quiet when you’re staring at that screen.”* (P03-Navy)

**Sub-theme 2.2: Evolution in the Nature and Intensity of Gambling**

*“I started betting bigger and more often, and I didn’t really notice it getting worse until it was too late.”* (P12-Army)

**Theme 3: Barriers and Supports for Help-Seeking During Service**

**Sub-theme 3.1: The Dichotomy of Military Support**

“*I think asking for help now is seen as a positive, whereas I think, in the military, with the stigma and the way people were, it was always seen as a negative. Then you were a liability. Probably why I didn't...*” (P13-Army)

*“You're trained to keep things to yourself. There’s no space for weakness and asking for help feels like that.”* (P12-Army)

**Sub-theme 3.2: Internalised Stigma and the Cycle of Financial Harm**

*“Everyone had loans or cards maxed out, it wasn’t just me. You kind of told yourself it was fine, like it was normal to be in debt.”* (P09-Navy)

*“You hit a point where you feel like you've messed up everything—money, family, work. Ending it starts to feel like a serious option.”* (P10-Army)

**Theme 4: Escalation of Gambling Post-Military Life, and Its Social Impact**

**Sub-theme 4.1: Civilian Transition and Escalation of Gambling**

*“It’s the escape from a mundane life. I struggle with boredom. So if life is boring and it's going to work, come home, go to work, come home, gambling gives you something to think about.”* (P13-Army)

*“When I came out and stuff, and I had lots of money, that went on alcohol, betting…”* (P03-Navy)

*“It was 24/7. You didn’t have to wait till the bookies opened. It was just always there, all the time.”* (P02-Navy)

**Sub-theme 4.2: The Impact of Gambling on Family and Social Relationships**

*“Within a short space of time I like lost my job, lost my house, got divorced, like it all came come tumbling down big time in one year”* (P04-RAF)

*“It affects your home life. I was getting home, snapping, irritable. Not because of her, but because I’d lost again. But she didn’t know that.”* (P12-Army)

*“I think people just got tired of me. I’d always be chasing money or talking about losses. It’s not exactly fun to be around, is it?”* (P09-Navy)
