# Supplementary material for: Qualitative exploration of gambling harm among UK veterans: normalisation, stigma and postservice escalation
Source: BMJ Open. 2026 Mar 25;16(3):e109458. doi: 10.1136/bmjopen-2025-109458 (PMC13034226; doi:10.1136/bmjopen-2025-109458)
Supplement: Supplementary file 3 [file bmjopen-2025-109458.reviewer_comments.pdf]

# PEER REVIEW HISTORY

BMJ Open publishes all reviews undertaken for accepted manuscripts. Reviewers are asked to complete a checklist review form and are provided with free text boxes to elaborate on their assessment. These free text comments are reproduced below.

## ARTICLE DETAILS

### Title (Provisional)

A Qualitative Exploration of Gambling Harm Among UK Veterans: Normalisation, Stigma, and Post-Service Escalation

### Authors

Dekel, Dana; Ekenna , Adanma; Engward, Hillary; Godier-McBard, Lauren R; Kay, Chris; Kersey, Thomas; Fossey, Matt; Dymond, Simon

---

## VERSION 1 - REVIEW

---

| | |
|---|---|
| **Reviewer** | **1** |
| **Name** | **Donnachie, Craig** |
| **Affiliation** | **University of Strathclyde, School of Psychological Sciences and Health** |
| **Date** | **07-Oct-2025** |
| **COI** | **None** |

---

Summary of paper

This qualitative study explored the lived experiences of UK Armed Forces veterans affected by gambling-related harms. This is an underserved population, and gambling-related harms are recognised as a growing public health concern. The findings presented illustrate how gambling harm among UK veterans is shaped by a complex interplay of cultural, emotional and institutional factors, including stigma etc. These findings are novel and make an important contribution to the field, including the need for culturally informed gambling harm reduction interventions and efforts, targeted at military veterans. The paper is well written. I have outlined my comments below, including areas that require further explication. I hope my comments are useful to the authors.

Comments

Introduction

Page 3, lines 11-14: It would be useful to describe what the term ‘gambling-related harm’ encapsulates, particularly when introducing in the first instance, especially for those uninitiated in this domain including explication of the specific dimensions (e.g. Langham et al

(2016) and Wardle et al (2018) frameworks) affecting resources, health/mental health and social relationships.

Page 3, lines 28-34: Can you describe more how specifically how cultural masculine ideals and constructions of masculinity/gender may influence high risk behaviour within this context, including with reference to wider literature.

Methods

Page 6, lines 15-28: Please could you clarify here if the study was exclusively inductive or if both deductive and inductive principles were employed.

Page 7, lines 11-12: Please can you elaborate on the how the trauma-informed approach was operationalised with regards to potentially distressing material. Also, how were researchers protected (e.g. opportunity for debriefing following interviews).

Page 7, lines 19-20: The topic guide does not appear in the supplementary files (only the checklist). Please upload and make available for the review process.

Page 6, lines 46-49: Please describe further how Reflexive Thematic Analysis was employed as opposed to Thematic Analysis (e.g. detailing the researcher's active, iterative engagement and interpretation including how their perspectives and assumptions guided analysis).

During the coding process it appears that both an inductive and deductive process was employed (could you clarify).

Could you further describe the wider team's involvement (e.g. did they read transcripts and/or code any of the transcripts?)

Page 7, lines 15-19: While principles of Grounded Theory are described it isn't clear how the key stages of RTA were employed (e.g. congruent with Braun and Clarke). Please can you describe how each of these systematic stages were operationalised.

Could illustrate how the COREQ framework as adhered to (e.g. via table) otherwise state further how this was operationalised.

Results

Page 7, lines 23-25: The following sentence pertains more to the Methods and suggest cutting as repeats process already described: "By the final interviews, no new codes or concepts were identified, confirming that data saturation had been reached".

Page 8, lines 14-15: You described thematic analysis but above you discussed reflexive thematic analysis (which are slightly distinct in accordance with Braun and Clarke's definitions). Keep consistent throughout.

Page 8, lines 17-20: Coding framework and the final overarching themes are usually discreet. In line with the analysis process, it would be useful to illustrate how the codes and themes evolved during analysis (including candidate themes etc).

General:

Inclusion of a thematic map or diagram outlining the main themes and sub-themes would be beneficial.

To streamline the findings and make more concise, it may be beneficial to include just one quote to illustrate each of the key points and include further rich/detailed examples in a table for example. This would enhance the readability and less reliance on the data to communicate the narrative.

Discussion

Page 19, lines 36-37: Thematic analysis (explicitly refer to RTA).

Page 22, lines 27-38: Much of the content is antithetical to the epistemological underpinnings of RTA outlined by Braun and Clarke (it is not clear why RTA was employed rather than traditional TA. Please provide further details on the approach employed and reflection, including strengths. Also, further consideration and reflection on the sample size being sufficient as it has been argued that N=15 is the minimum required for TA.

Page 23, lines 9-17: Can you provide further details on possible implications, including how the findings presented may inform future interventions (e.g. targeting stigma) as well as importance of future means of managing distress (including suicide ideation) given the findings presented.

---

**Reviewer** 2

**Name** Metcalf, Olivia

**Affiliation** Phoenix Centre for Posttraumatic Mental Health, Department of Psychiatry, University of Melbourne

**Date** 22-Dec-2025

**COI** None

---

This is a well-written and important paper. I have the following suggestions:

1. Please add the evidence gap into the abstract

2. Several of the quotes and sub-themes can be moved to tables to shorten the length of the manuscript.

3. There is a comment in the manuscript

4. Sub-theme 4.1 has already been extensively discussed in the literature (e.g., citation 9). It is not clear how and what new information this study adds to our understanding of transition as a time of vulnerability for gambling.

5. The introduction is clear and well-set up, however, the discussion is fairly long and draws in much of the background literature already established. In general, this paper simply

confirms what we already know about gambling and veterans, using qualitative data. I am not sure that is strong enough. I had trouble discerning what your findings specifically added- what are the clinical, policy, and research implications of these findings? What does this study specifically add, beyond the other quantitative studies?

---

## VERSION 1 - AUTHOR RESPONSE

Reviewer: 1

**We thank Reviewer 1 for their careful reading of the manuscript and for their constructive and encouraging feedback. We are grateful for the positive assessment of the study's contribution and for the detailed suggestions, which have helped us to strengthen the conceptual clarity, methodological transparency, and presentation of the findings. We respond to each comment below.**

Introduction
Page 3, lines 11-14: It would be useful to describe what the term 'gambling-related harm' encapsulates, particularly when introducing in the first instance, especially for those uninitiated in this domain including explication of the specific dimensions (e.g. Langham et al (2016) and Wardle et al (2018) frameworks) affecting resources, health/mental health and social relationships.

**We have expanded the opening definition of gambling-related harm to clarify that it encompasses harms extending beyond gambling behaviour itself, including impacts on financial resources, mental and physical health, and social and relational functioning. We now explicitly reference established conceptual frameworks (Langham et al., 2016; Wardle et al., 2018) to situate our use of the term within the wider public health literature, (p.3):**

Gambling-related harm is a significant public health issue globally, with far-reaching consequences for individuals, families, and communities (Wardle et al., 2024). Gambling-related harm is understood as a multidimensional concept that extends beyond gambling behaviour itself to encompass harms to financial resources, physical and mental health, social and family relationships, and occupational functioning (Langham et al., 2015; Wardle et al., 2024). These frameworks emphasise that harm may occur across a continuum of gambling involvement and can affect both individuals who gamble and those around them. In the United Kingdom, Armed Forces veterans are disproportionately affected, exhibiting substantially higher rates of gambling related harm compared to the general population (Dighton et al., 2018). Research indicates that veterans are approximately seven times more likely to experience problem gambling (PG), defined as a pattern of gambling behaviour that disrupts personal, familial, or occupational functioning, with 43% of veterans reporting gambling issues compared to just 6.5% of non-veterans (Harris et al., 2023)

Page 3, lines 28-34: Can you describe more how specifically how cultural masculine ideals and constructions of masculinity/gender may influence high risk behaviour within this context, including with reference to wider literature.

**We have revised the Introduction to further specify how dominant constructions of military masculinity shape risk-taking, emotional suppression, and responses to distress. In particular, we clarify how stoicism, endurance, and self-reliance are institutionally valued, while emotional**

**disclosure and help-seeking may be stigmatised, and how this may normalise high-risk behaviours, (p.3):**

Military life fosters a distinctive behavioural culture shaped by hierarchy, routine, and risk exposure. Social norms within this environment often promote emotional suppression and competitiveness, reinforcing a masculine ideal that can normalise high-risk behaviours and deprioritise emotional wellbeing (Randles & Finnegan, 2022). Within military institutions, dominant constructions of masculinity emphasise stoicism, endurance, and self-reliance, while expressions of vulnerability and emotional distress are frequently stigmatised as incompatible with the ideal soldier identity (Fox & Pease, 2012). This cultural framework may inadvertently desensitise personnel to signs of emerging harm and discourage early recognition of problematic gambling. Fox and Pease (2012), further argue that these norms shape how distress is interpreted and managed, often privileging avoidance, emotional containment, and risk-taking over help-seeking or early intervention. Additionally, many veterans face identity confusion, social isolation, and economic instability during reintegration, all of which may exacerbate susceptibility to gambling problems (Adams et al., 2021).

Methods

Page 6, lines 15-28: Please could you clarify here if the study was exclusively inductive or if both deductive and inductive principles were employed.

**We have clarified that both data collection and analysis were inductive in orientation. Specifically, we now state that the pilot phase did not generate pre-defined analytic categories, that the interview schedule was intentionally non-directive and participant-led, and that coding was inductive, with existing literature informing analytic sensitivity rather than constituting a deductive framework. These clarifications have been added to the Data Collection and Data Analysis sections (p.6-7):**

Page 7, lines 11-12: Please can you elaborate on the how the trauma-informed approach was operationalised with regards to potentially distressing material. Also, how were researchers protected (e.g. opportunity for debriefing following interviews).

**We have expanded the Data Collection section to clarify how a trauma-informed approach was operationalised in practice. Specifically, we now describe how participant choice, pacing, and control were prioritised during interviews, how distress was monitored and managed, and how participants were debriefed and signposted to support. We have also clarified the arrangements for researcher support and debriefing following potentially distressing interviews, (pp.6-7):**

***Data collection***

Prior to formal data collection, a pilot stage was conducted with three veterans from our lived experience panel. This process informed several refinements to the interview schedule, including adjustments to language, tone, and the ordering of questions to ensure greater clarity, accessibility, and sensitivity. The pilot did not generate pre-defined analytic categories but was used to refine the phrasing and flow of questions to support open, participant-led discussion. It also confirmed the relevance of key topic areas and helped establish a trauma-informed approach suited to discussing

potentially distressing material. A trauma-informed approach was operationalised throughout data collection by prioritising participant safety, choice, and control. Participants were reminded that they could pause, decline to answer questions, or stop the interview at any point without providing a reason. Interview pacing was guided by participants, with sensitive topics approached flexibly rather than in a fixed sequence. The interviewer remained attentive to signs of distress and checked in with participants when discussing potentially triggering material. All participants were debriefed at the end of the interview and provided with signposting to relevant support services.
Fourteen one-to-one semi-structured interviews were conducted between September 2024 and May 2025. All participants who consented to take part completed the interview. Each interview lasted approximately 45 to 60 minutes and was conducted via confidential Zoom meetings. Each participant took part in a single interview; no repeat interviews were conducted. Interviews followed a semi-structured format to allow for flexibility while eliciting rich, in-depth data (Bryman et al., 2021). The interview schedule was intentionally broad and non-directive, enabling participants to shape the focus and depth of discussion and allowing unanticipated issues to emerge. ~~Broad, ~~O~~o~~pen-ended questions were used to explore participants' lived experiences with gambling harm, including their gambling history, pathways into gambling, motivating factors, control strategies, help-seeking behaviours during and after military service, and the impact of online gambling (interview schedule, ~~-~~supplementary file 1).

Prior to the interviews, all participants were provided with an information sheet outlining the background and aims of the study. They were given time to review the materials, ask questions, and provide written informed consent. Upon consent, interviews were audio-recorded and later transcribed verbatim. Transcripts were not returned to participants for comment or correction, in order to minimise participant burden and maintain confidentiality. All interviews were conducted individually by a trained member of the research team (DD), who had no prior relationship with the participants. Participants were compensated for their time with a £20 voucher. Due to the small and potentially identifiable nature of the sample, direct identifiers such as names, sex or age have been omitted. To support researcher wellbeing, the interviewer had access to regular opportunities for informal debriefing within the research team following interviews that involved potentially distressing material.

Ethical approval for the study was granted by the Swansea University School of Psychology Research Ethics Committee (Approval number: 1 2024 8399 8380).

***Data Analysis***
The interview data were analysed using thematic analysis as outlined by Braun and Clarke (2006), and further elaborated in their reflexive approach (Braun & Clarke, 2021) following an inductive, data-driven approach (Thomas, 2006). The reflexive orientation acknowledged the active role of the researcher in interpreting and constructing meaning from the data, while the inductive approach allowed patterns and themes to emerge directly from the data rather than being imposed by existing theoretical frameworks. This was essential given the exploratory nature of the research and the diversity within the sample. Thematic analysis was particularly suitable for this study, as it facilitated a nuanced understanding of varied perspectives without assuming a single explanatory theory (Braun & Clarke, 2022). The COREQ checklist was used to guide transparent reporting across study design, data collection, analysis, and reflexivity, with relevant items addressed throughout the Methods section.~~The findings are presented in line with the COREQ checklist for reporting qualitative research~~ (Tong et al., 2007)

Analysis followed the phases outlined by Braun and Clarke, including familiarisation with the data, inductive coding, iterative development of candidate themes, review and refinement of themes, and reflexive interpretation throughout the analytic process.

To maintain confidentiality, each participant was assigned a unique numerical code, and all identifying details were removed or anonymised during transcription. Transcripts were reviewed carefully to ensure anonymity and completeness before analysis. ~~A preliminary~~ Initial coding ~~framework~~ was ~~initially~~ led ~~developed~~ by the lead researcher (DD), and was inductive in orientation, with codes generated through close engagement with the data rather than through the application of pre-defined categories. Existing literature informed the researcher's analytic sensitivity but did not constitute a deductive coding framework ~~drawing on both existing literature and inductively derived insights from the data~~ (Thomas, 2006). As analysis progressed, initial codes were grouped into broader themes through iterative comparison and reflection. To enhance the trustworthiness of the analysis, and in accordance with the criteria outlined by Elo et al., (2014), several measures were taken to ensure methodological rigour. Credibility was strengthened through deep immersion in the data, involving multiple close readings of transcripts, line-by-line coding, and the use of ongoing memo-writing. Reflexivity was embedded throughout the analytic process, with the lead researcher engaging in ongoing reflection on how their assumptions, positionality, and prior engagement with the topic shaped interpretation. Collaborative input from members of the research team further supported the dependability and confirmability of the findings. Consistent with a reflexive thematic analysis approach, team discussions were used to support reflexive dialogue and analytic refinement rather than to achieve consensus coding or inter-rater reliability. Themes were reviewed and refined through regular team discussions, ensuring clarity, coherence, and alignment with the study's aims.

NVivo (version 22) software was used to support the organisation of transcripts, systematic coding, and memo development throughout the analytic process. Iterative comparison across transcripts was used to explore similarities and differences within the dataset; references to constant comparison draw on general analytic principles rather than indicating adherence to grounded theory as a methodological framework ~~A constant comparative method, drawing on principles of grounded theory~~ (Glaser, 1965). ~~, was employed to explore similarities and differences across the dataset.~~ To ensure that the findings remained grounded in participants' lived experiences, the results are illustrated with rich, representative quotations.

Page 7, lines 19-20: The topic guide does not appear in the supplementary files (only the checklist). Please upload and make available for the review process.

**Thank you for noting this. The interview schedule has now been uploaded and made available as a supplementary file**.

Page 6, lines 46-49: Please describe further how Reflexive Thematic Analysis was employed as opposed to Thematic Analysis (e.g. detailing the researcher's active, iterative engagement and interpretation including how their perspectives and assumptions guided analysis).

**This point is addressed in the expanded Data Analysis section described above. We have clarified how reflexive thematic analysis was operationalised, including the researcher's active and iterative engagement with the data, the role of reflexive memo-writing, and how researcher assumptions and positionality informed interpretation, in line with Braun and Clarke's reflexive approach (revised pp.6-7; see response to previous comment).**

**Addressing three below comments jointly:**

During the coding process it appears that both an inductive and deductive process was employed (could you clarify).

Could you further describe the wider team's involvement (e.g. did they read transcripts and/or code any of the transcripts?)

Page 7, lines 15-19: While principles of Grounded Theory are described it isn't clear how the key stages of RTA were employed (e.g. congruent with Braun and Clarke). Please can you describe how each of these systematic stages were operationalised.

**These points are addressed in the revised Data Analysis section. We now clarify that coding was inductive in orientation, with existing literature informing analytic sensitivity rather than constituting a deductive framework; describe the role of the wider team in supporting reflexive dialogue rather than consensus coding; and explicitly outline how the phases of reflexive thematic analysis were operationalised in line with Braun and Clarke (revised pp.6-7).**

Could illustrate how the COREQ framework as adhered to (e.g. via table) otherwise state further how this was operationalised.

**We have clarified how the COREQ framework was operationalised by specifying that it was used to guide transparent reporting across study design, data collection, analysis, and reflexivity, with relevant items addressed throughout the Methods section (revised p.7).**

The COREQ checklist was used to guide transparent reporting across study design, data collection, analysis, and reflexivity, with relevant items addressed throughout the Methods section.~~The findings are presented in line with the COREQ checklist for reporting qualitative research~~ (Tong et al., 2007).

Results
Page 7, lines 23-25: The following sentence pertains more to the Methods and suggest cutting as repeats process already described: "By the final interviews, no new codes or concepts were identified, confirming that data saturation had been reached".

**We agree and have removed this sentence from the Results section. Considerations relating to sample adequacy and analytic sufficiency are addressed in the Methods section**

Page 8, lines 14-15: You described thematic analysis but above you discussed reflexive thematic analysis (which are slightly distinct in accordance with Braun and Clarke's definitions). Keep consistent throughout.

**We have revised the manuscript to ensure consistent use of the term *reflexive thematic analysis* throughout, in line with Braun and Clarke's definitions, and removed ambiguous references to thematic analysis where these could cause confusion.**

Page 8, lines 17-20: Coding framework and the final overarching themes are usually discreet. In line with the analysis process, it would be useful to illustrate how the codes and themes evolved during analysis (including candidate themes etc).

**We have revised the Results section to ensure consistent use of *reflexive thematic analysis* and added a brief analytic signposting paragraph under the "Reflexive thematic analysis" subheading. This clarifies how inductive codes were iteratively developed into candidate and final themes, with the thematic table presenting the outcome of this reflexive analytic process (p.9):**

**Reflexive Thematic Analysis**

Reflexive tThematic analysis generated four major themes through an iterative analytic process involving inductive coding, development of candidate themes, and ongoing refinement through reflexive interpretation. Four major themes were identified: (1) Cultural Normalisation of Gambling in UK and Military Contexts; (2) Gambling Preferences and Motivations During Military Service; (3) Barriers and Supports for Help-Seeking During Service; and (4) Escalation of Gambling Post-Military Life and Its Social Impact. The coding framework, including themes and sub-themes, is are summarised in Table 2 and illustrated with anonymised participant quotes throughout the Results section., providing a clear description of the coding structure used in the analysis.

General:
Inclusion of a thematic map or diagram outlining the main themes and sub-themes would be beneficial.

**Thank you for this suggestion. We considered the inclusion of a thematic diagram to visually represent the main themes and sub-themes. However, during revision we found that a separate diagram did not add substantive analytic value beyond the existing thematic table and accompanying narrative, which already clearly present the structure and relationships of the findings. We therefore retained the table and strengthened the Results signposting to ensure clarity and accessibility**

To streamline the findings and make more concise, it may be beneficial to include just one quote to illustrate each of the key points and include further rich/detailed examples in a table for example. This would enhance the readability and less reliance on the data to communicate the narrative.

**Thank you for this suggestion. We agree that clarity and readability are important and have therefore streamlined the Results section by reducing repetition across quotations. In response, we have removed 17 quotations where they reiterated points already illustrated elsewhere, while retaining those that contributed distinct analytic insight or captured key experiential nuances. These changes are visible in the tracked-changes version of the revised manuscript.**

**We consider participant voices to be central to the aims of this qualitative study. To preserve transparency and enable interested readers to access the fuller dataset, the removed quotations have been collated and made available as a supplementary file.**

Discussion
Page 19, lines 36-37: Thematic analysis (explicitly refer to RTA).

**We have addressed this by ensuring consistent and explicit reference to reflexive thematic analysis throughout the manuscript, including in the Discussion section (see response to comments above).**

Page 22, lines 27-38: Much of the content is antithetical to the epistemological underpinnings of RTA outlined by Braun and Clarke (it is not clear why RTA was employed rather than traditional TA. Please provide further details on the approach employed and reflection, including strengths. Also, further consideration and reflection on the sample size being sufficient as it has been argued that N=15 is the minimum required for TA.

**We have revised the opening of the Discussion to explicitly situate the analysis within a reflexive thematic analysis framework and to clarify the epistemological positioning of the study. We now state why reflexive thematic analysis was selected and how theoretical frameworks are used as sensitising concepts rather than deductive models, in line with Braun and Clarke's guidance. (p.20):**

This study explored the lived experiences of gambling-related harm among UK Armed Forces veterans, with a focus on how military culture, psychological factors, and institutional structures shaped gambling trajectories, barriers to help-seeking, and long-term social impacts. Reflexive tThematic analysis generated four interrelated themes, each of which reflects the complex interplay between cultural normalisation, psychological vulnerability, structural stigma, and life transitions. These findings build on and extend current understanding of gambling harm in military contexts, offering critical insights into the social processes that render such harm both pervasive and hidden. Consistent with a reflexive thematic analysis approach (Braun & Clarke, 2021, 2023), the themes presented do not claim to represent objective truths or universal mechanisms, but rather reflect the researchers' interpretive engagement with participants' situated accounts. Existing theoretical frameworks are used in the Discussion as sensitising concepts to deepen interpretation, rather than as explanatory models imposed during analysis. This positioning aligns with the epistemological foundations of reflexive thematic analysis, which emphasise reflexivity, context, and meaning making over prediction or theory verification.

**Regarding sample size, this was acknowledged and reflected upon as a limitation in the original manuscript. In the revised version, we have added further clarification to explicitly note that numerical minimum thresholds are not a requirement within reflexive thematic analysis, where adequacy is determined by information power, analytic depth, and reflexive engagement rather than participant counts (pp. 23-24):**

**Strengths, Limitations, and Implications**

This study offers rich, first-person accounts of gambling harm in a group underrepresented in the literature. By centering the voices of veterans, it provides insight into not only the risk factors but also the social and institutional contexts that shape harm trajectories. A key strength lies in its qualitative design, which allowed for in-depth exploration of complex and sensitive themes that are often overlooked in survey-based research. Nonetheless, reflexive thematic analysis is not without limitations. Its flexibility, while advantageous for exploring subjective experiences, may also introduce the possibility of researcher influence (Jason & Glenwick, 2016). Although inductive coding aims to reduce bias, a researcher's background and assumptions can still shape how themes are constructed (Joffe & Yardley, 2003). However, rather than being viewed solely as a source of bias, this subjectivity can be understood as a valuable analytic resource (Varpio et al., 2021). The active role of the researcher in interpreting and constructing meaning is recognised as part of the analytic process (Braun & Clarke, 2023). Collaborative discussion of codes and themes supported reflexive dialogue, helping to

incorporate diverse perspectives and enhance analytic rigour.~~Collaborative development of the coding framework helped incorporate diverse perspectives and enhance analytic rigour~~.

This sample, while diverse in-service background, was small and self-selecting, potentially limiting generalisability. Although the sample size was guided by the concept of information power (Malterud et al., 2016), data saturation was also observed during coding, with no new codes or concepts emerging in the final interviews (Guest et al., 2006; Saunders et al., 2018). While some authors have proposed numerical minimum sample sizes for thematic analysis, such thresholds are not a requirement within reflexive thematic analysis, where analytic adequacy is determined by information power, analytic depth, and reflexive engagement rather than participant counts (Braun & Clarke, 2021). All participants had already identified themselves as having experienced gambling-related harm, which may not reflect those with subclinical or unacknowledged problems. Only one participant identified as female, limiting the ability to explore potential gendered differences in gambling experiences, help-seeking behaviour, or institutional responses. Additionally, while the study included veterans from three service branches, intersectional perspectives (e.g., related to ethnicity, gender, or LGBTQ+ status) were limited and warrant further investigation.

Page 23, lines 9-17: Can you provide further details on possible implications, including how the findings presented may inform future interventions (e.g. targeting stigma) as well as importance of future means of managing distress (including suicide ideation) given the findings presented.

**Thank you for highlighting the importance of this point. We agree that the links between gambling harm, emotional distress, and suicidal ideation represent a critical aspect of the findings. In response, we have expanded the implications section to provide more detailed consideration of how the findings may inform future interventions, including stigma reduction, integrated gambling harm and mental health support, and the importance of addressing distress and suicidal ideation identified in participants' accounts. (p.24):**

Despite these limitations, the findings suggest several important directions for policy and practice. First, routine gambling screening should be introduced across the Armed Forces, including pre-enlistment, active service, and discharge processes. Evidence-based tools tailored to military populations (Rayner et al., 2025) must be implemented consistently. Second, stigma reduction efforts need to be embedded not only in education and training, but also within disciplinary systems to ensure that help-seeking does not lead to professional penalty. Finally, tailored post-discharge interventions should address the unique psychological, financial, and relational challenges veterans face, with a particular focus on transitions and identity loss. The findings also highlight the importance of interventions that explicitly address the links between gambling harm, emotional distress, and suicidality. Several participants described suicidal ideation in the context of escalating debt, shame, and isolation. Future interventions should therefore integrate gambling harm support with mental health and suicide prevention pathways. This includes training military and veteran-facing services to recognise gambling-related distress as a potential suicide risk factor, and ensuring clear, non-punitive referral routes to psychological and crisis support. Trauma-informed, confidential approaches that promote early disclosure may be critical in preventing escalation to crisis.

**********************************************

Reviewer 2:

**We thank Reviewer 2 for their careful reading of the manuscript and for their thoughtful and**

**constructive feedback. We are grateful for the positive assessment of the paper's clarity and importance, as well as for the critical reflections regarding originality, contribution, and the articulation of implications. These comments prompted us to substantially refine the Discussion, sharpen the paper's analytic contribution, and clarify how the findings extend existing evidence. In response, we have condensed sections of the Discussion, strengthened the focus on interpretive and process-level insights, and more explicitly articulated the clinical, policy, and research implications of the findings. We respond to each comment below.**

Comments to the Author:

This is a well-written and important paper. I have the following suggestions:

1. Please add the evidence gap into the abstract

**We have added a clear statement of the evidence gap to the abstract, specifying the limited qualitative research on veterans' lived experiences of gambling harm and how this study addresses that gap:**

**Objective:** This study explored the lived experiences of UK Armed Forces veterans affected by gambling-related harm. It examined how military culture, institutional practices, and life transitions shaped gambling behaviours, barriers to help-seeking, and the long-term impact on wellbeing. While quantitative research has documented elevated gambling-related harm among veterans, little qualitative work has examined how veterans themselves understand, experience, and navigate gambling harm across military and post-military contexts.

2. Several of the quotes and sub-themes can be moved to tables to shorten the length of the manuscript.

**Thank you for this suggestion. We agree that clarity and readability are important and have therefore streamlined the Results section by reducing repetition across quotations. In response, we have removed 17 quotations where they reiterated points already illustrated elsewhere, while retaining those that contributed distinct analytic insight or captured key experiential nuances. These changes are visible in the tracked-changes version of the revised manuscript.**

**We consider participant voices to be central to the aims of this qualitative study. To preserve transparency and enable interested readers to access the fuller dataset, the removed quotations have been collated and made available as a supplementary file.**

3. There is a comment in the manuscript

**Thank you for bringing this to our attention, the comment has now been removed.**

4. Sub-theme 4.1 has already been extensively discussed in the literature (e.g., citation 9). It is not clear how and what new information this study adds to our understanding of transition as a time of vulnerability for gambling.

**Thank you for this observation. We agree that transition from service has been widely identified as a period of vulnerability in the existing literature. Our intention was not to restate this finding, but**

**to extend it by showing how transition operates as a critical step within a broader narrative of gambling harm escalation.**

**In the revised manuscript, we have clarified this contribution in the Discussion by explicitly articulating how participants described transition as a turning point through which gambling intensified and subsequently contributed to downstream consequences such as escalating debt, relationship breakdown, social isolation, and severe psychological distress, including suicidal ideation. Rather than positioning transition as a standalone risk factor, the revised discussion emphasises how gambling functioned during this period and how it preceded deeper escalation of harm.**

The post-military transition emerged ~~was~~ as a critical period during which gambling often escalated. The ~~sudden~~ loss of military routine, ~~purpose, and~~ identity and structure left many participants vulnerable to boredom, emotional strain, and isolation. Consistent with Fry et al. (2024) and Adams et al.(2021), gambling became more frequent, immersive, and emotionally charged after discharge, particularly as participants sought ways to manage unstructured time and loneliness. The accessibility of online gambling further intensified this shift, enabling private, continuous engagement without external scrutiny ~~these findings underscore how structural and emotional instability following discharge can increase risk behaviours, particularly in the absence of tailored support systems. Several participants reported that gambling became more frequent, immersive, and emotionally charged after leaving service, serving as a mechanism to cope with loneliness or manage unstructured time. The accessibility of online gambling further intensified this shift, offering constant and private opportunities to gamble without external scrutiny. Reflecting broader concerns in the literature on digital gambling risks~~ (Gainsbury, 2015; Wardle et al., 2024). ~~regarding the immersive nature of digital gambling platforms and their potential to facilitate compulsive play, particularly among those experiencing distress or disconnection.~~

While transition has been widely recognised as a period of vulnerability, this study sheds light on the lived processes through which gambling becomes embedded during this period. Participants described using gambling to manage boredom, identity loss, and emotional dysregulation following discharge. Their accounts indicate that transition often marked a turning point that preceded deeper escalation, including worsening debt, relationship breakdown, social isolation, and severe psychological distress.

**We have also ensured that the implications section clearly reflects how these escalation trajectories inform clinical, policy, and prevention responses, including the need for integrated gambling harm, mental health, and suicide prevention pathways (p.24):**

Despite these limitations, the findings suggest several important directions for policy and practice. First, routine gambling screening should be introduced across the Armed Forces, including pre-enlistment, active service, and discharge processes. Evidence-based tools tailored to military populations (Rayner et al., 2025) must be implemented consistently. Second, stigma reduction efforts need to be embedded not only in education and training, but also within disciplinary systems to ensure that help-seeking does not lead to professional penalty. Finally, tailored post-discharge interventions should address the unique psychological, financial, and relational challenges veterans face, with a particular focus on transitions and identity loss. The findings also highlight the importance of interventions that explicitly address the links between gambling harm, emotional distress, and suicidality. Several participants described suicidal ideation in the context of escalating debt, shame, and isolation. Future interventions should therefore integrate gambling harm support with mental health and suicide prevention pathways. This includes training military and veteran-facing services to recognise gambling-related distress as a potential suicide risk factor, and ensuring clear, non-punitive

referral routes to psychological and crisis support. Trauma-informed, confidential approaches that promote early disclosure may be critical in preventing escalation to crisis.

**This study therefore contributes insight into the lived mechanisms through which known risk periods translate into cumulative harm, providing contextual depth that is not captured by prevalence-focused studies alone.**

5. The introduction is clear and well-set up, however, the discussion is fairly long and draws in much of the background literature already established. In general, this paper simply confirms what we already know about gambling and veterans, using qualitative data. I am not sure that is strong enough. I had trouble discerning what your findings specifically added- what are the clinical, policy, and research implications of these findings? What does this study specifically add, beyond the other quantitative studies?

**Thank you for this important and thoughtful comment. We agree that it was necessary to sharpen the contribution of the Discussion and to make explicit what this study adds beyond the existing literature.**

**In response, we have substantially condensed the Discussion, reducing repetition of background literature and strengthening the focus on what the findings uniquely contribute. Specifically, we revised each thematic subsection to prioritise interpretation of participants' accounts rather than re-describing established evidence, and we clarified how the findings extend, rather than merely confirm, prior work.**

**The revised Discussion now makes clear that this study adds value in several ways that are not captured in quantitative research alone. First, it provides process-level insight into *how* gambling harm develops and escalates across the service life course, including how gambling functions as an emotionally regulated coping strategy within military culture, rather than simply co-occurring with distress. Second, it documents institutional and relational consequences that are difficult to capture in survey data, including relationship breakdown, loss of contact with children, concealment driven by stigma, and trajectories leading to severe psychological distress and suicidality. Third, it demonstrates how transition from service operates as a turning point within a wider escalation pathway, rather than a standalone risk factor, showing how boredom, identity loss, and emotional dysregulation interact to deepen harm over time.**

**We have also strengthened the clinical, policy, and research implications section to clearly articulate how these findings inform practice. In particular, the revisions emphasise the need for integrated gambling harm, mental health, and suicide prevention pathways; non-punitive, stigma-informed support structures within military systems; and early intervention approaches that recognise gambling-related distress as a marker of broader psychosocial risk. These implications are now explicitly grounded in participants' accounts rather than inferred from prevalence data alone.**

**Taken together, the revisions clarify that the contribution of this study lies not in restating known associations, but in providing contextual, experiential, and relational depth that explains how and why gambling harm unfolds in veterans' lives, thereby informing more responsive and targeted intervention strategies (pp.20-25):**

**Discussion**

This study explored the lived experiences of gambling-related harm among UK Armed Forces veterans, with a focus on how military culture, psychological factors, and institutional structures shaped gambling trajectories, barriers to help-seeking, and long-term social impacts. Reflexive t~~T~~hematic analysis generated four interrelated themes, each of which reflects the complex interplay between cultural normalisation, psychological vulnerability, structural stigma, and life transitions. These findings build on and extend current understanding of gambling harm in military contexts, offering critical insights into the social processes that render such harm both pervasive and hidden. Consistent with a reflexive thematic analysis approach (Braun & Clarke, 2021, 2023), the themes presented do not claim to represent objective truths or universal mechanisms, but rather reflect the researchers' interpretive engagement with participants' situated accounts. Existing theoretical frameworks are used in the Discussion as sensitising concepts to deepen interpretation, rather than as explanatory models imposed during analysis. This positioning aligns with the epistemological foundations of reflexive thematic analysis, which emphasise reflexivity, context, and meaning-making over prediction or theory verification.

**Military and Civilian Cultures as Sites of Gambling Normalisation**

Gambling was described as embedded across the life course of the UK culture, from early exposure in civilian contexts to routine engagement during military service. This reflects findings on gambling's cultural normalisation in both society (McGee, 2020; Newall et al., 2019; Wardle et al., 2024) and within military settings (Champion et al., 2022; Milton et al., 2020). ~~Our ~~P~~p~~articipants portrayed ~~military ~~gambling during service as widespread and institutionally tolerated, driven by boredom, downtime routines, and peer influence.

The social acceptability of gambling was reinforced by group cohesion and a lack of scrutiny, with behaviours framed as morale-building rather than risky. These ~~findings ~~aligns with ~~studies ~~research showing ~~how ~~that military environments ~~treat ~~often position gambling as ordinary ~~and ~~or even beneficial (Dighton et al., 2018; Dighton et al., 2025; Milton et al., 2020), obscuring harm and complicating help-seeking (Champion et al., 2025; Vana et al., 2025). ~~When ~~Within ~~situated within ~~rigid hierarchies and ~~a ~~cultures of emotional control, gambling may become normalised, concealed and difficult to detect, particularly when embedded in everyday institutional ~~it aligns with broader institutional norms and remains embedded in everyday ~~practices (Randles & Finnegan, 2022; Wardle et al., 2024).

**Psychological Coping and Escalation During Service**

Participants described using fast-paced, non-strategic gambling as a way to detach from stress, emotional strain, and cognitive overload during military service. Rather than being motivated by financial gain or excitement, gambling functioned as a means of switching off, managing mood, and achieving emotional distance during downtime or after demanding shifts.~~Participants described using repetitive, non-strategic gambling to detach from stress and emotion. These forms of gambling were described as fast-paced, immersive, and requiring minimal cognitive effort, making them conducive to switching off from work demands, emotional strain, or routine stressors. Rather than seeking monetary gain or excitement, participants described using gambling as a way to achieve psychological distance, manage mood, and blunt emotional reactivity during periods of downtime or after difficult shifts.~~ This pattern aligns with research on avoidance-based emotion regulation in institutional settings characterised by high performance demands, restricted autonomy, and norms of emotional control~~This use of gambling as a coping tool aligns with Gross's (1998) emotion regulation model, which distinguishes between antecedent-focused strategies (modifying the situation or one's~~

perception of it before an emotional response occurs) and response-focused strategies (modifying the emotional response once it has been generated), and with research on maladaptive regulation in institutional settings which shows that environments characterised by rigid hierarchies, restricted autonomy, and high performance demands can foster avoidance-based coping strategies such as substance use or repetitive, immersive activities (Dowling et al., 2015; Gross, 1998; Redmond et al., 2015).

Gambling was used to manage mood, avoid distress, and create emotional distance. This aligns with the escape/relief pathway described in Blaszczynski and Nower's (2002) Pathways Model, which characterises a subgroup of gamblers who engage in gambling to alleviate negative emotional states such as anxiety, stress, or depression. In this context, gambling functions as an avoidant coping mechanism, providing temporary relief or distraction from adverse emotions and life stressors. Participants' accounts of using fast-paced, repetitive gambling activities to "switch off" resonate with this framework, reflecting how gambling can serve as a form of emotional regulation when other, healthier coping strategies are unavailable or inaccessible.

While trauma-related explanations may offer one relevant lens (Grubbs & Chapman, 2019; Vana et al., 2025), participants' accounts in this study point to a broader affective process. Gambling was actively selected for its capacity to induce immersion, time-loss, and dissociative states, particularly through electronic gambling machines and fixed-odds betting terminals, which have been shown to facilitate these effects our findings indicate a more general affective pattern: gambling served as an accessible strategy for emotional numbing and cognitive disengagement, particularly within a culture that emphasises control, discipline, and performance. In particular, electronic gambling machines and fixed-odds betting terminals have been shown to facilitate time-loss, immersion, and dissociative states, making them especially suitable for escape-oriented gambling (Griffiths, 1993; Murch & Clark, 2021; Schüll, 2012). In this context, gambling functioned as an avoidant coping strategy, consistent with the escape/relief pathway described by Blaszczynski and Nower (2002), providing temporary relief from distress rather than entertainment or reward.

Participants also described a gradual and often unnoticed shift from casual to compulsive gambling. This escalation was facilitated by steady income, limited immediate financial consequences during service, and a cultural emphasis on self-reliance and stoicism that discouraged disclosure or help-seeking. Similar structural enablers have been identified in serving personnel (Champion et al., 2022). Many participants described a gradual and often unnoticed shift from casual to compulsive gambling. This transition was typically enabled by a combination of steady and often disposable income, limited institutional oversight, and a cultural ethos that emphasised personal responsibility and self-reliance. These conditions created an environment in which gambling could escalate unchecked. Similar patterns were observed in the Champion et al. (2022) study with serving RAF personnel, where high income and downtime were identified as key enablers of persistent gambling. Military norms around stoicism, emotional restraint, and self-sufficiency further reinforced avoidance-based coping, making it more likely that gambling would be used to regulate distress rather than trigger support-seeking. This aligns with previous research showing how such norms can contribute to maladaptive behaviours, including emotional suppression, substance use, and withdrawal However, participants' narratives in the present study illustrate how these conditions interacted to normalise gambling as a privately damaging but socially acceptable outlet for distress, allowing harm to develop largely unchecked. This extends existing evidence by illuminating the lived mechanisms through which cultural norms and institutional structures sustain gambling escalation during service (Coleman et al., 2017; Heward et al.,

2024; McElheran et al., 2024; Sharp et al., 2015). This analysis clarifies how gambling becomes normalised as a functional coping strategy within military life, rather than merely a co-occurring behaviour. ~~Within this context, gambling became a socially acceptable yet privately damaging outlet—one that flourished in a culture where vulnerability was often equated with weakness.~~

**Stigma, Silence, and Structural Barriers to Help-Seeking**

Stigma emerged as a key barrier to disclosure. Participants ~~shared deeply personal accounts of~~ described withholding help-seeking ~~disclosure~~ due to fear of reputational damage, disciplinary action, or forced discharge. While some recalled nominal encouragement to seek support through welfare services or chaplaincy, these ~~systems~~ pathways were often perceived as inconsistent, inaccessible, or actively risky ~~to engage with~~. The coexistence of formal support structures ~~official support pathways~~ and informal sanctions created a climate of mistrust and ambiguity.

These findings align with existing research on stigma and support in military contexts ~~This reflects existing literature on stigma and support in military contexts~~ (Sharp et al., 2015; Silvestrini & Chen, 2023), and highlights how gambling is ~~often~~ frequently overlooked or mishandled. Rather than initiating care, disclosure could trigger punitive responses. This contradiction reflects a form of structural stigma, where institutional messaging about support is undermined by its consequences (Coleman et al., 2017; Heward et al., 2024; Sharp et al., 2015). In this ~~environment~~context, gambling harm was not only stigmatised but also framed as a professional liability. This insight adds nuance to ~~current~~ debates on military mental health support ~~in military settings~~ by showing that barriers to care arise not from the absence of support, but from the risks attached to using it. ~~support systems may be undermined not by their absence but by the institutional consequences tied to accessing them.~~

Internalised stigma intensified these challenges. Participants described feeling pressured to appear "sorted" and emotionally self-sufficient, echoing ~~broader~~ military norms ~~around~~ of discipline, control, and resilience (Coleman et al., 2017; Heward et al., 2024). ~~These patterns~~Such norms are consistent with ~~research~~ evidence on avoidant coping in military personnel, where emotional suppression and hyper-independence ~~are valued traits that may hinder timely~~may delay intervention (McElheran & Stelnicki, 2024). ~~Some~~ Several participants ~~described~~ reported significant emotional deterioration, including suicidal ideation illustrating ~~. These experiences show~~ how stigma can escalate distress ~~, delay help,~~ and deepen harm. Given the ~~presence of~~normalisation of gambling within military life, systems must be prepared for ~~the possibility of~~ addiction-related harm. Support ~~should~~ responses need to be consistent, non-punitive, and culturally responsive; ~~to the cultural environment that shapes behaviour.~~ Without this, gambling-related distress is likely to remain concealed and unaddressed. ~~personnel may remain silent, and gambling harm will continue to be overlooked.~~

**Post-Service Life as a Locus of Escalation and Isolation**

The post-military transition emerged ~~was~~ as a critical period during which gambling often escalated. The ~~sudden~~ loss of military routine, ~~purpose, and~~ identity and structure left many participants vulnerable to boredom, emotional strain, and isolation. Consistent with Fry et al. (2024) and Adams et al.(2021), gambling became more frequent, immersive, and emotionally charged after discharge, particularly as participants sought ways to manage unstructured time and loneliness. The accessibility of online gambling further intensified this shift, enabling private, continuous engagement without external scrutiny ~~these findings underscore how structural and emotional instability following discharge can increase risk behaviours, particularly in the absence of tailored support systems. Several~~

~~participants reported that gambling became more frequent, immersive, and emotionally charged after leaving service, serving as a mechanism to cope with loneliness or manage unstructured time. The accessibility of online gambling further intensified this shift, offering constant and private opportunities to gamble without external scrutiny. Reflecting broader concerns in the literature on digital gambling risks~~ (Gainsbury, 2015; Wardle et al., 2024). ~~regarding the immersive nature of digital gambling platforms and their potential to facilitate compulsive play, particularly among those experiencing distress or disconnection.~~

While transition has been widely recognised as a period of vulnerability, this study sheds light on the lived processes through which gambling becomes embedded during this period. Participants described using gambling to manage boredom, identity loss, and emotional dysregulation following discharge. Their accounts indicate that transition often marked a turning point that preceded deeper escalation, including worsening debt, relationship breakdown, social isolation, and severe psychological distress.

Financial mismanagement compounded this escalation. Many participants lacked experience managing civilian expenses and described using gambling as a financial escape, often drawing on discharge payments or credit in secrecy. The relational consequences were substantial, with participants reporting relationship breakdown, loss of contact with children, and social withdrawal, alongside suicidal ideation (Kalischuk et al., 2006; Marionneau & Nikkinen, 2022). ~~was also a recurring theme. Participants often lacked experience with budgeting or managing civilian expenses, making them more susceptible to using gambling as a financial escape. Some drew on lump-sum payments received at discharge to fund their gambling, while others turned to credit or loans in secrecy. The emotional and relational costs of this escalation were significant. Participants described how concealment, debt, and shame contributed to the breakdown of intimate relationships, loss of contact with children, and withdrawal from friends. Kalischuk et al. reported similar consequences, where relational breakdown and social isolation are prominent features of escalating behaviour, alongside relational disruption and family strain. Participants in the present study described how these dynamics were compounded by indebtedness, shame, and suicidal ideation .~~Attempts at self-regulation, such as self-exclusion or betting limits, were commonly ineffective, aligning with evidence that individual control strategies often fail without systemic support ~~Despite using tools such as self-exclusion or gambling limits, most reported limited long-term success, which aligns with wider evidence showing that personal regulation strategies often fail without systemic support~~ (Livingstone et al., 2019).

**Strengths, Limitations, and Implications**

This study offers rich, first-person accounts of gambling harm in a group underrepresented in the literature. By centering the voices of veterans, it provides insight into not only the risk factors but also the social and institutional contexts that shape harm trajectories. A key strength lies in its qualitative design, which allowed for in-depth exploration of complex and sensitive themes that are often overlooked in survey-based research. Nonetheless, reflexive thematic analysis is not without limitations. Its flexibility, while advantageous for exploring subjective experiences, may also introduce the possibility of researcher influence (Jason & Glenwick, 2016). Although inductive coding aims to reduce bias, a researcher's background and assumptions can still shape how themes are constructed (Joffe & Yardley, 2003). However, rather than being viewed solely as a source of bias, this subjectivity can be understood as a valuable analytic resource (Varpio et al., 2021). The active role of the researcher in interpreting and constructing meaning is recognised as part of the analytic process (Braun & Clarke, 2023). Collaborative discussion of codes and themes supported reflexive dialogue, helping to incorporate diverse perspectives and enhance analytic rigour.~~Collaborative development of the coding framework helped incorporate diverse perspectives and enhance analytic rigour.~~

This sample, while diverse in-service background, was small and self-selecting, potentially limiting generalisability. Although the sample size was guided by the concept of information power (Malterud et al., 2016), data saturation was also observed during coding, with no new codes or concepts emerging in the final interviews (Guest et al., 2006; Saunders et al., 2018). While some authors have proposed numerical minimum sample sizes for thematic analysis, such thresholds are not a requirement within reflexive thematic analysis, where analytic adequacy is determined by information power, analytic depth, and reflexive engagement rather than participant counts (Braun & Clarke, 2021). All participants had already identified themselves as having experienced gambling-related harm, which may not reflect those with subclinical or unacknowledged problems. Only one participant identified as female, limiting the ability to explore potential gendered differences in gambling experiences, help-seeking behaviour, or institutional responses. Additionally, while the study included veterans from three service branches, intersectional perspectives (e.g., related to ethnicity, gender, or LGBTQ+ status) were limited and warrant further investigation.

Despite these limitations, the findings suggest several important directions for policy and practice. First, routine gambling screening should be introduced across the Armed Forces, including pre-enlistment, active service, and discharge processes. Evidence-based tools tailored to military populations (Rayner et al., 2025) must be implemented consistently. Second, stigma reduction efforts need to be embedded not only in education and training, but also within disciplinary systems to ensure that help-seeking does not lead to professional penalty. Finally, tailored post-discharge interventions should address the unique psychological, financial, and relational challenges veterans face, with a particular focus on transitions and identity loss. The findings also highlight the importance of interventions that explicitly address the links between gambling harm, emotional distress, and suicidality. Several participants described suicidal ideation in the context of escalating debt, shame, and isolation. Future interventions should therefore integrate gambling harm support with mental health and suicide prevention pathways. This includes training military and veteran-facing services to recognise gambling-related distress as a potential suicide risk factor, and ensuring clear, non-punitive referral routes to psychological and crisis support. Trauma-informed, confidential approaches that promote early disclosure may be critical in preventing escalation to crisis.

**Conclusion**

This study provides a detailed account of how gambling harm is shaped by cultural, institutional, and psychological factors within and beyond military service. Gambling is not merely an individual problem, but a socially constructed and institutionally sustained phenomenon that emerges from specific high-risk, occupational environments. Despite its prevalence and impact, gambling is not currently recognised as a priority issue within UK military or veteran health systems, resulting in a lack of screening, data collection, and tailored support. To prevent harm, interventions must go beyond individual education and include systemic changes to how gambling is understood, addressed, and supported across the Armed Forces and veteran care systems. Efforts to normalise disclosure, provide culturally competent care, and disrupt harmful norms around self-reliance and emotional suppression are essential if we are to reduce gambling harm and its devastating consequences among UK veterans. By foregrounding veterans' lived accounts, this study adds explanatory depth to existing prevalence research, illustrating how known risk factors translate into cumulative harm across military and post-military life.

---

## VERSION 2 - REVIEW

**Reviewer** 1

**Name** Donnachie, Craig

**Affiliation** University of Strathclyde, School of Psychological Sciences and Health

**Date** 13-Feb-2026

**COI**

---

I am satisfied that the authors have thoroughly and comprehensively addressed all of my comments, illustrated clearly in their response letter and revised manuscript. I have no further points other than to congratulate the team on their excellent work.

---

**Reviewer** 2

**Name** Metcalf, Olivia

**Affiliation** Phoenix Centre for Posttraumatic Mental Health, Department of Psychiatry, University of Melbourne

**Date** 26-Jan-2026

**COI**

---

Thank you for addressing my comments. In re-reading this, I realized as a non-British person, the following terms don't mean anything to non-British readers:

Derby

Grand National

penny pusher

High street

Wetherspoons

NAAFI

fruit machine

tenner

quid

RAF

SSAFA

---

## VERSION 2 - AUTHOR RESPONSE

**Response to Reviewer**

Thank you for highlighting the reviewer's concern regarding UK-specific idiomatic and cultural references. We agree that several terms may not be immediately clear to an international readership.

In response, we have retained all participant quotations in their original form to preserve data integrity, and have added concise contextual clarifications in brackets at first mention of relevant terms. These additions are provided either within the narrative text or immediately following the relevant quotation.

Examples include clarification of culturally specific references such as "High Street" (town's main commercial street), "NAAFI" (UK Armed Forces retail and leisure organisation operating on military bases), "SSAFA" (UK Armed Forces welfare charity), and colloquial monetary terms such as "tenner" (£10) and "a few quid" (a few pounds).

These amendments can be seen clearly in the tracked version of the revised manuscript.

We hope these revisions enhance accessibility for international readers while preserving the authenticity of participants' accounts.
